# Biophysical versus machine learning models for predicting rectal and skin temperatures in older adults

**DOI:** 10.1016/j.jtherbio.2025.104078

**Published:** 2025-02-22

**Authors:** Connor Forbes, Alberto Coccarelli, Zhiwei Xu, Robert D. Meade, Glen P. Kenny, Sebastian Binnewies, Aaron J.E. Bach

**Affiliations:** a School of Information and Communication Technology, Griffith University, Gold Coast, Australia; b Zienkiewicz Institute for Modelling, AI and Data, Mechanical Engineering Department, Faculty of Science and Engineering, Swansea University, Swansea, UK; c School Medicine and Dentistry, Griffith University, Gold Coast, Australia; d Cities Research Institute, Griffith University, Gold Coast, Australia; e Human and Environmental Physiology Research Unit, School of Human Kinetics, University of Ottawa, Ottawa, Canada; f Harvard T.H. Chan School of Public Health, Harvard University, Boston, United States; g School of Health Sciences and Social Work, Griffith University, Gold Coast, Australia

**Keywords:** Aged, Body temperature, Extreme heat, Heat stress disorders, Theoretical models

## Abstract

This study compares the efficacy of machine learning models to traditional biophysical models in predicting rectal (T_re_) and skin (T_sk_) temperatures of older adults (≥60 years) during prolonged heat exposure. Five machine learning models were trained on data using 4-fold cross validation from 162 day-long (8–9h) sessions involving 76 older adults across six environments, from thermoneutral to heatwave conditions. These models were compared to three biophysical models: the JOS-3 model, the Gagge two-node model, and an optimised two-node model. Our findings show that machine learning models, particularly ridge regression, outperformed biophysical models in prediction accuracy. The ridge regression model achieved a Root-Mean Squared Error (RMSE) of 0.27 °C for T_re_, and 0.73 °C for T_sk_. Among the best biophysical models, the optimised two-node model achieved an RMSE of 0.40 °C for T_re_, while JOS-3 achieved an RMSE of 0.74 °C for T_sk_. Of all models, ridge regression had the highest proportion of participants with T_re_ RMSEs within clinically meaningful thresholds at 70% (<0.3 °C) and the highest proportion for T_sk_ at 88% (<1.0 °C), tied with the JOS-3 model. Our results suggest machine learning models better capture the complex thermoregulatory responses of older adults during prolonged heat exposure. The study highlights machine learning models’ potential for personalised heat risk assessments and real-time predictions. Future research should expand upon training datasets, incorporate more dynamic conditions, and validate models in real-world settings. Integrating these models into home-based monitoring systems or wearable devices could enhance heat management strategies for older adults.

## 1 Introduction

Humans deploy both autonomic and behavioural regulation in order to maintain homeothermy and preserve essential physiological functions. Skin temperature plays a crucial role in this process, as it influences, in-part, behavioural thermoregulation (Vargas et al., 2018) and is the site of heat exchange between the body and the surrounding environment. However, prolonged and compensable heat strain leading to relatively small perturbations in core temperature (a rise <1 °C) can lead to severe health consequences in at risk populations (Faunt et al., 1995; Loughnan et al., 2010; Meade et al., 2024c). The measurement of core temperature is invasive, often requiring devices such as rectal thermometers or ingestible sensors, which limits its practicality in many settings. The accurate prediction of core and skin temperatures could help better define individual risk, early warning, and allow for intervention to prevent heat-related illnesses and injuries during prolonged exposure to indoor overheating caused by hot weather or heatwaves.

The consequences of heat-related morbidity and mortality are not equally shared amongst society. Older adults are among the most vulnerable (Bach et al., 2024; Meade et al., 2020). This vulnerability stems from a combination of factors, including but not limited to, age-related impairments in thermoregulation, reduced adaptive capacity and social support, a higher prevalence of chronic health conditions, and associated medications that can hinder heat loss capacity (Kenny et al., 2019, 2024).

Various physics and data-driven models and standards have been developed to predict heat strain across heat exposed populations, particularly in architectural, occupational, military, and athletic settings (An et al., 2023; Lundgren-Kownacki et al., 2017; Mantzios et al., 2024; Potter et al., 2017; Tan et al., 2021; Yermakova et al., 2022). In recent years, computer models for predicting the risk of heat strain in older persons have garnered renewed interest (Coccarelli et al., 2018; Deng et al., 2018; Itani et al., 2020; Ji et al., 2022; Joshi et al., 2022; Kang and Wang, 2019; Ou et al., 2023; Rida et al., 2014; Zhao et al., 2020) likely due to their disproportionate risk of mortality and morbidity during heat events, which are forecast to increase with climate change (IPCC, 2023). Improving these predictive models could aid in developing timely interventions and personalised alerts to message heat risk at the individual level and minimise heat-related morbidity and mortality among older populations (Hansen et al., 2011). Such predictions would serve as a proactive mechanism for behaviour change before an individual’s heat risk reaches more critical thresholds. The environment where older adults experience heat — primarily within their home (Kenny et al., 2019, 2024; Klepeis et al., 2001) — can differ substantially from outdoor conditions (Gupta et al., 2021; Henderson et al., 2022; Sakka et al., 2012). A real-world example might see the coupling of accurate physiological predictions with real-time household environmental sensors, allowing for smart-home devices to provide advice for when to implement personal cooling strategies or relocate to cooler environments as the risk of heat exposure increases (Oberai et al., 2025). This pre-emptive approach is especially vital in urban areas prone to heat islands or during extreme heat events (i.e., prolonged duration of extreme heat), where traditional weather forecasts may not adequately capture the personalised risk to vulnerable populations (Kenny et al., 2024).

Thermophysiological (or physics-based) models can be categorised into several types: one-node models, which treat the whole body as a lumped system; multi-node models like the Gagge model (Gagge et al., 1986) and JOS-3 (Takahashi et al., 2021) which divide the body into multiple homogenised compartments, and multi-element continuum models such as (Coccarelli et al., 2018; Kang and Wang, 2019), which allow to reconstruct the temperature space distribution within each element of the body. These models may account for various exertional and environmental heat stress and rely on detailed biophysical parameters to account for the conditions encountered during any (in)activity. The predictive accuracy of the current physiological models is hindered by the fact that these methodologies are often validated by considering conditions which do not necessarily reflect extreme heat events, due to their short exposures (<120 min) and/or relatively low environmental temperatures (<37 °C) (Coccarelli et al., 2018; Itani et al., 2020; Ji et al., 2022; Ou et al., 2023; Rida et al., 2014; Takahashi et al., 2021). Recently, it has been demonstrated that it takes up to 4–6 h to achieve stable core temperatures during simulated heatwave exposures in older adults (Meade et al., 2024d). Consequently, exposures of shorter duration may not provide sufficient time to reach thermal equilibrium, potentially underestimating the true core temperature responses of participants. Additionally, while these physiological models may account for the participants’ age, this is generally achieved through ‘one-size-fits-all’ adjustments to model parameters and/or equations. However, the physiological response to prolonged heat exposure may be extremely variable between older adults and this undermines the standard approach. Assuming sufficient valid training data, machine learning models represent a promising alternative for addressing this shortfall by being capable to account for the vast inter-participant variability. Machine learning has previously shown promising results in thermal comfort models (Kocaman et al., 2024). However, there is little work done on the prediction of human body temperature based on ambient conditions, especially in an older cohort of heat-vulnerable individuals.

In the presented work, we introduce and assess different data-driven/machine learning models for core and skin temperatures estimation, for both individual and group responses in resting older adults (≥ 60 years) exposed to six ambient environments ranging from thermoneutral to heatwave conditions.

## 2 Methods

### 2.1 Development of machine learning temperature prediction models

We considered five different machine learning model types including: linear regression, ridge regression, recurrent neural networks (RNN), long-short term memory networks (LSTM), and gated recurrent units (GRU). There are two overarching architectures of machine learning models investigated here, point-wise prediction models and sequence prediction models.

The point-wise prediction models, specifically linear and ridge regression, forecast body temperatures (both skin and core) for the next minute based solely on the current body temperatures, environmental temperature, and participant characteristics. These models utilise minute-by-minute data to maximise the training dataset, thereby enhancing predictive performance. The estimated function maps temperature changes between consecutive time points without considering historical data beyond the immediate previous minute. Both regression models were implemented using the scikit-learn Python library (Pedregosa et al., 2011).

On the other hand, time-sequence predictions models, such as the RNN, LSTM, and GRU attempt to predict the entire sequence of temperatures at once. These models take into account the temporal dependencies between consecutive minutes by updating internal state variables based on the input features and the previous state. This acts as a form of memory and allows capture of long-term patterns in the data. For example, they can account for cumulative heat gain over an extended period (hours to days), which is critical for understanding how prolonged exposure to heat affects body temperature. These models were implemented using the Keras Python machine learning library (Chollet, 2015).

The sequence prediction models shared a common architecture. The input sequence was first processed by the respective recurrent layer (GRU, LSTM, or RNN). This recurrent layer’s unit size was a key hyperparameter, with values of 8, 16, 32, and 64 evaluated. The optimal unit size for each model type was determined by selecting the architecture that yielded the lowest RMSE on the validation set. For the LSTM and RNN models, a unit size of 16 was determined to result in the lowest RMSE, while for the GRU model the optimal unit size was 32. Following the recurrent layer, the output was passed through a Time Distributed Dense layer with 8 units and a linear activation function, regularized using an L2 kernel penalty of 0.01. To mitigate overfitting, a Dropout layer with a rate of 0.2 was introduced. Finally, another Time Distributed Dense layer with an output shape matching the prediction target (in this case, 2 units for core and skin temperature) was applied. Model training for each architecture was conducted for a maximum of 500 epochs, incorporating early stopping with a patience of 10 epochs based on validation loss. Due to computational constraints, an exhaustive exploration of the entire architecture and hyperparameter space was not feasible.

**Fig. 1:**
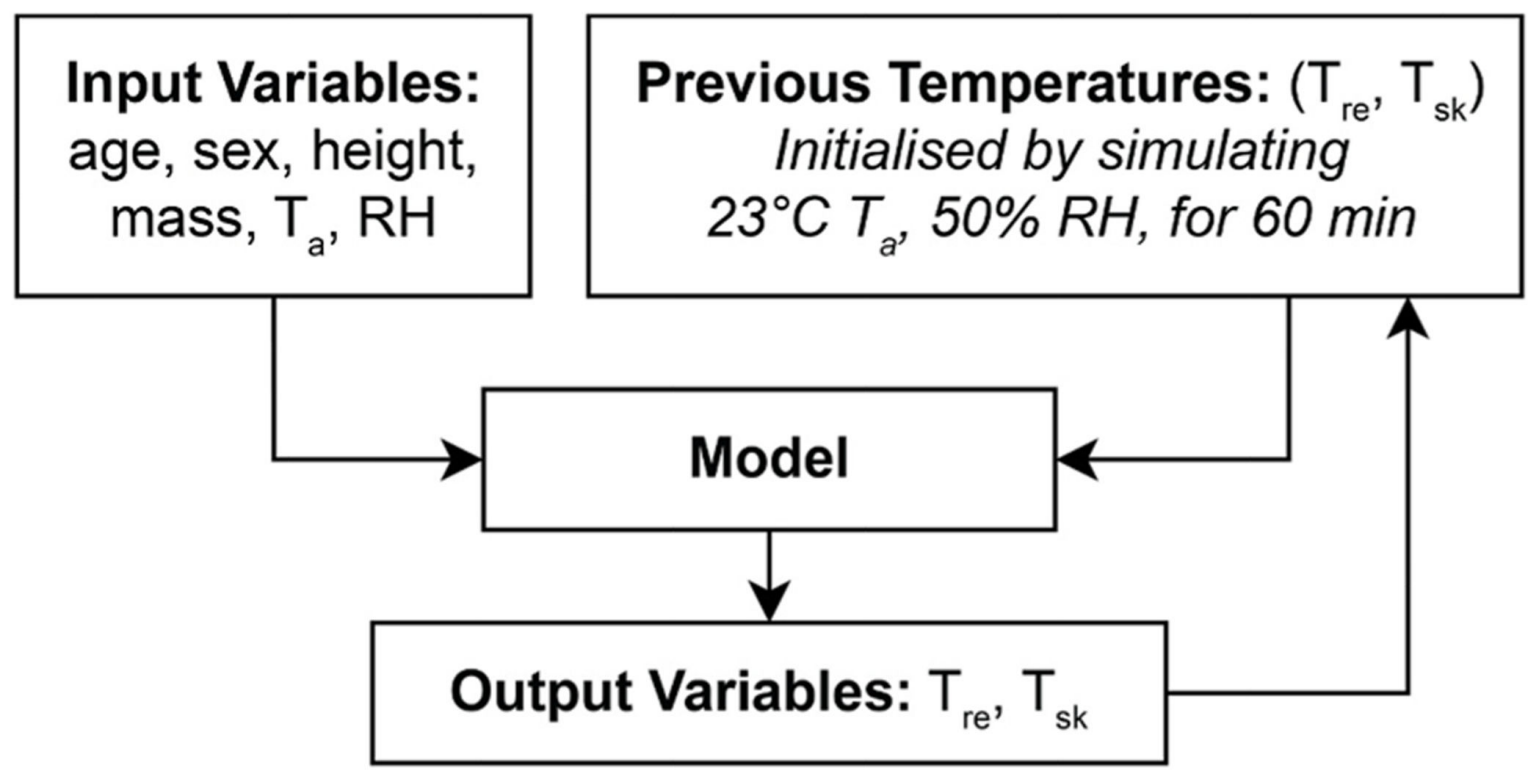
Data flow diagram for validation of the point-wise models (linear regression and ridge regression). Input variables include sex, age, body height, body mass, ambient temperature (T_a_), and relative humidity (RH). Output variables are rectal temperature (T_re_) and mean skin temperature (T_sk_).

To personalise the model predictions, we considered the available recorded participant features—sex, age (years), height (cm), mass (kg), ambient temperature (°C), and relative humidity (RH, %)—as inputs for all models. The output of the models were rectal temperature (°C) and mean skin temperature (°C) at specific time points. We used rectal temperature as the index of core temperature as this is what was measured in the experimental data. Mean skin temperature was also present in the experimental data and calculated based on the following weightings: 7% forehead, 17.5% right scapula, 17.5% upper left chest, 7% upper right arm, 7% right forearm, 5% left hand, 19% right anterior thigh, and 20% left calf (ISO, 2004). Since the point-wise models lack internal memory, they also included the skin and rectal temperatures from the previous time point as inputs. To initialise these values, the models were run with ambient conditions set to 23 °C and 50% RH for 120 min. During validation, the previous temperatures were based on the model’s output from the previous time step and were fed back iteratively. This means that the model’s predictions from one time step were used as inputs for the next time step, creating a feedback loop that simulates a sequential dependency. This iterative process for validation of the models is illustrated in Fig. 1.

We employed 4-fold cross-validation to train and evaluate our models. In this approach, the dataset was first partitioned into four equal-sized, distinct subsets (or “folds”). Each fold, in turn, served as the validation set, while the remaining three folds were combined to form the training set. This means each fold used 75% of participants (57 individuals) for training and 25% (19 individuals) for validation. This process was repeated four times, ensuring that each data point was used for validation exactly once. Cross-validation provides a more robust estimate of model performance compared to a single train/validation split, particularly when dealing with limited data, by reducing the risk that the specific composition of the training or validation set will overly influence the results. The aggregate results from these four iterations were used to assess each model’s overall performance over the entire dataset. The performance of each model in each fold is presented in the Supplement Tables S1–S3

**Fig. 2:**
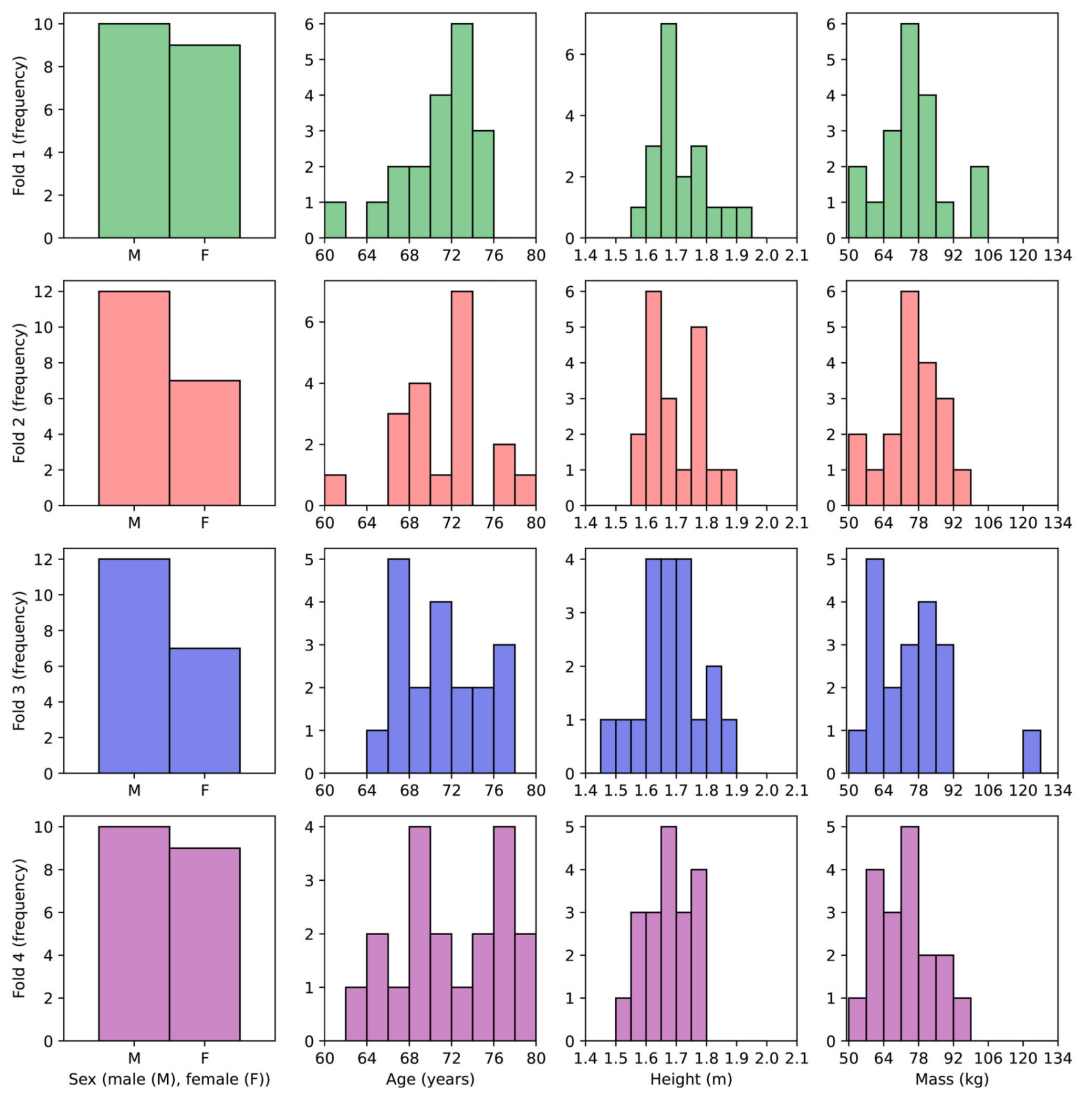
Distribution of characteristics between training and validation set.

To create these folds, we randomly selected distinct individuals, identified by unique participant IDs, across all experimental conditions. A distinct individual is defined as a single participant who may appear in multiple experimental conditions but is represented by the same unique participant ID. Thus, all data from a single individual was kept within the same fold to prevent data leakage and ensure an unbiased evaluation. The spread of participants across conditions and characteristics of the participants for each fold are presented in Table 1 and Fig. 2 respectively.

During training and validation, all input and output variables were normalised to a range between zero and one using a min-max scaler. A min-max scaler transforms data by scaling each feature to a given range (in this case, 0 to 1) based on the minimum and maximum values of the feature. This is represented in Equation (1):

(1) $$X_{\text{scaled}\;}=\frac{X-X_{\min}}{X_{\max}-X_{\min}}$$

where, *X*_min_ is the minimum value for that feature present in the dataset; *X*_max_ is the maximum value for that feature in the dataset; *X* is the variable to be scaled; and *X*_scaled_ is the resulting normalised value (between 0 and 1).

When evaluating the predictions, these normalised output values were transformed back to their original scale. This inverse transformation allows us to obtain the actual skin and rectal temperatures from the normalised data and is represented by Equation (2):

(2) $$X=X_{\text{scaled}\;}•\left(X_{\max}-X_{\min}\right)+X_{\min}$$

Each of the machine learning models evaluated in this paper are reported in further detail below.

### 2.2 Linear regression

In linear regression, the goal during training is to minimise the residual sum of squares (RSS) between the predicted and observed outputs (rectal and mean skin temperature) (Rencher and Schaalje, 2007). By adopting a linear regression model, the core (rectal) and mean skin temperatures can be expressed as a linear combination of different factors:

(3) $${\hat{y}}_{\text{tre}}=\beta_{1}x_{1}+\beta_{2}x_{2}+\ldots +\beta_{8}x_{8}+\beta_{c}$$

(4) $${\hat{y}}_{\text{tsk}}=\beta_{1}^{\prime }x_{1}+\beta_{2}^{\prime }x_{2}+\ldots +\beta_{8}^{\prime }x_{8}+\beta_{c}^{\prime }$$

where, *β*_*j*_ and $\beta_{j}^{\prime }$ represent the coefficients for each feature in the respective models; *x*_1_ to *x*_8_ are the considered features: sex (0 = male, 1 = female), age (years), body height (cm), body mass (kg), ambient temperature (°C), relative humidity (%RH), previous rectal temperature (°C), previous mean skin temperature (°C). The previous rectal and mean skin temperatures are taken from the previous time step, which corresponds to the previous minute; *ŷ*_tre_ is the predicted rectal temperature (°C); *ŷ*_tsk_ is the predicted mean skin temperature (°C); and *β*_*c*_ and $\beta_{c}^{\prime }$ are the intercepts for each model.

RSS is calculated as per Equation (5):

(5) $$\text{RSS}=\sum_{i=1}^{n}{\left(y_{i,tmp}-{\hat{y}}_{i,tmp}\right)}^{2}$$

where, *y*_*i*,*tmp*_ being the temperature *tmp* ∈ {tsk, tre} for each time point *i*. The final values for the coefficients after training can be found in the Supplementary material.

### 2.3 Ridge regression

Ridge regression adds a penalty factor (*α* = 1) to linear regression that penalises the size of the coefficients using the sum of their squares (L2 regularisation) (Hoerl and Kennard, 2000). The idea behind this is to reduce overfitting by shrinking the coefficients of less important features towards zero, while also preventing the model from relying too heavily on any single strong feature (e.g. ambient temperature, previous rectal/mean skin temperature). This approach balances the influence of multiple coefficients, leading to a more robust and generalisable model. The RSS Equation (6) for ridge regression is:

(6) $${\text{RSS}}_{R}=\overset{n}{\underset{i=1}{\sum }}{\left(y_{i,tmp}-{\hat{y}}_{i,tmp}\right)}^{2}+\alpha \overset{8}{\underset{j=1}{\sum }}\beta_{j}^{2}$$

### 2.4 Recurrent neural networks (RNN)

RNN are a class of artificial neural networks designed to process sequential data, such as time series or natural language. Unlike traditional feedforward neural networks, RNN incorporate a feedback mechanism that allows them to maintain a form of “memory” of previous inputs (Rumelhart et al., 1986). The key feature of RNN is the hidden state, which acts as a form of memory. At each time step, the network updates this hidden state based on the current input and the previous hidden state. This allows the network to maintain context over time, which is crucial for tasks like predicting future values in a time series or understanding the meaning of words in a sentence based on previous words.

In a fully connected RNN, the current context at time *t*, calculated as *h*_*t*_ = tanh (*g*_*t*_), is passed as input to the context calculation for time *t* + 1, where:

(7) $$g_{t}=W_{g}\cdot h_{t-1}+U_{g}\cdot x_{t}+b_{g}$$

and, *W*_*g*_ is the weight matrix for the hidden layer; *h*_*t*−1_ is the hidden layer vector at time *t* − 1; *U*_*g*_ is the weight matrix for the input vector; *x*_*t*_ is the input vector at time *t*; and *b*_*g*_ is the bias term. The output vector is calculated as *ŷ*_*t*_ = *W*_*ŷ*_⋅*h*_*t*_ + *b*_*ŷ*_.

### 2.5 Long-short term memory (LSTM)

In many applications, phenomena may have both short-term and long-term dependencies. Standard RNN often struggle with such long-term dependencies due to the vanishing gradient problem during training. LSTM enhance RNN with long-term retention of memory by introducing a forget gate *f*_*t*_, input gate *i*_*t*_, and output gate *o*_*t*_ (Hochreiter and Schmidhuber, 1997). The gates control which information is retained in a cell *c*_*t*_:

(8) $$c_{t}={\tilde{c}}_{t}⊙i_{t}+c_{t-1}⊙f_{t}$$

with ${\tilde{c}}_{t}=tanh\left(g_{t}\right),\left\{i_{t},f_{t},o_{t}\right\}=\sigma \left(g_{t}\right)$, where *σ* is the *sigmoid* function, *h*_*t* -1_ = *o*_*t*–1_ ⊙ tanh (*c*_*t* -1_), and ⊙ is the matrix element-wise product (also known as the Hadamard product). This formulation allows the network to selectively remember or forget information over long sequences, making LSTM particularly useful for tasks requiring long-term memory, such as analysing long time series data or understanding context in large text corpora.

### 2.6 Gated recurrent units (GRU)

GRU are a simplified version of LSTM, designed to achieve similar performance with reduced computational complexity. GRU simplify LSTM by merging the forget and input gates into an update gate *z*_*t*_ and introducing a reset gate *r*_*t*_ to combine the cell state and current context (Cho et al., 2014). With {*z*_*t*_, *r*_*t*_} = *σ* (*g*_*t*_) and *σ* being the *sigmoid* function, each hidden state vector *h*_*t*_ at time *t* is calculated as:

(9) $$h_{t}={\tilde{h}}_{t}⊙z_{t}+h_{t-1}⊙z_{t}$$

(10) $${\tilde{h}}_{t}=\tanh(W_{\tilde{h}}•\left(r_{t}⊙h_{t-1}\right)+U_{\tilde{h}}•x_{t}+b_{\tilde{h}}$$

### 2.7 Participant data and model training

For model training and evaluation, we used a dataset of 162 day-long exposure sessions involving 76 unique participants, sourced from the University of Ottawa (Meade et al., 2023a, 2023b, 2024a). These participants were exposed to a temperature and humidity-controlled test chamber under six different ambient conditions, and their rectal temperature and mean skin temperature were measured. The ambient conditions were:

- **Condition 1**–40 °C at 9% RH for 540 min;
- **Condition 2**–40 °C at 9% RH for 225 min, then 23 °C at 50% RH for 120 min, then 40 °C at 9% RH for 195 min;
- **Condition 3**–36 °C at 45% RH for 480 min;
- **Condition 4**–31 °C at 45% RH for 480 min;
- **Condition 5**–26 °C at 45% RH for 480 min;
- **Condition 6**–22 °C at 45% RH for 480 min.

We included 76 (44 male; 32 female) participants aged ≥60 years in our study. Of these, we randomly assigned each participant into one of four folds for cross-validation. In each fold of the cross-validation process, the model was trained on the participants not included in that fold and evaluated on those who were. One participant was excluded from condition 3 due to incomplete data. Some participants appeared across multiple conditions. The distribution of participants for each condition is shown in Table 1. The participant IDs assigned to each fold were:

- **Fold 1:** 24, 28, 43, 50, 57, 62, 66, 68, 71, 72, 75, 79, 83, 86, 90, 93, 94, 95, 97
- **Fold 2:** 22, 25, 26, 32, 34, 35, 45, 48, 54, 55, 59, 67, 69, 70, 74, 76, 85, 91, 98
- **Fold 3:** 21, 27, 30, 37, 42, 46, 47, 49, 53, 61, 63, 65, 73, 78, 81, 82, 87, 92, 96
- **Fold 4:** 23, 29, 33, 36, 38, 39, 40, 41, 44, 52, 56, 58, 60, 64, 77, 80, 84, 88, 89

Fig. 2 shows that across all folds, most participant characteristics (age, body height, and body mass) are evenly distributed. Each fold displays a slight bias towards males. To ensure this bias does not impact results, additional error quantification was performed on the male and female groups. For the sex attribute, a value of one indicates female while zero indicates male.

### 2.8 Comparison to existing biophysical models

To identify existing biophysical models for potential comparison with our machine learning approach, we conducted a targeted literature search in PubMed. This search, while not a comprehensive systematic review, aimed to locate any models designed specifically to predict core and skin temperatures of older persons (≥60 years) under heat stress. Inclusion criteria for biophysical models were as follows: (1) derived from original research published in peer-reviewed English-language journals between January 1980 and October 2022; (2) designed or adjusted for individual older adult (≥60 years) outputs, incorporating age-related decline in thermoregulatory function; (3) applicable across diverse racial/ethnic groups; (4) capable of modelling hyperthermia through passive and/or exertional means in an air medium; (5) validated against reliable measures of core and/or skin temperatures from human experiments; and (6) publicly available (open-source) or accessible for evaluation against our dataset through collaboration with the model developers. Out of 3031 studies identified, 98 were removed due to being duplicate studies. After screening titles and abstracts, 202 were identified as relevant. Full-text screening further narrowed this down to 10 potential studies. An additional model which was published following our search (Wang et al., 2023), left us with 11 published older person biophysical models.

From the 11 studies, the model by Coccarelli et al. (2018) was not capable of simulating such long-term scenarios (up to 540 min) as used in our study. Morris et al. (2021) implemented heat balance equations without core/skin temperature outputs. Two models (Hirata et al., 2015; Kang and Wang, 2019) were not shared by the authors for this evaluation. Two others (Davoodi et al., 2018; Wang et al., 2023) were unable to be included after initial agreement for simulation from the developers before contact ceased. A single model used in two studies (Itani et al., 2020; Rida et al., 2014) was shared, but excluded as it produced population-level rather than individual predictions. Of two open-source models that were sequential developed from one another (JOS-2: Kobayashi and Tanabe, 2013; JOS-3: Takahashi et al., 2021), only the latest JOS-3 model (Takahashi et al., 2021) was included. One model was replicated with author assistance – an optimised two-node model for older persons (Ji et al., 2022) – and included in the evaluation. For these reasons, only these two models (Ji et al., 2022; Takahashi et al., 2021) designed for predicting hyperthermia in older persons were included. The seminal two-node model created by Gagge et al. (1986), was also included as a comparator to establish the baseline performance of a model that was not created for specifically for older people. For the remainder of this paper, the three comparator models will be referred to by the last name of the first author for simplicity (two-node, Gagge; optimised two-node, Ji; JOS-3, Takahashi).

### 2.9 Model evaluation

To assess the performances of the models, a 4-fold cross-validation strategy was used. This was to ensure that the machine learning/regression models were not over-fitted to the training data. To initialise the initial rectal and mean skin temperatures, all models were simulated in ambient conditions at 23 °C and 50% RH for 120 min.

The two-node models (Gagge and Ji) were based off the two-node model provided in ‘pythermalcomfort’ (Tartarini and Schiavon, 2020), a Python package which integrates various thermal comfort models (including Gagge’s two-node model). For condition 1 and 2, the clothing value (clo) of males was set to 0.15 to represent wearing: underwear, shorts and slippers. For all other participants’ conditions the clo value was set 0.23 to represent wearing: underwear, shorts, slippers and a T-shirt (Parkinson and De Dear, 2017). We set the metabolic rates (MET) of participants to be 1.3 kcal kg^−1^⋅h^−1^ to represent seated rest (Mansoubi et al., 2015). The full list of input parameters for the two-node models is given in Table 2. The mean radiant temperature was assumed equal to dry bulb air temperature, as the validation data was collected in indoor environments and the models are intended for indoor heatwave risk assessment in older adults.

For the Takahashi model, the same inputs were used with slight adjustments to accommodate the model’s requirements. Specifically, instead of using a MET value of 1.3, an equivalent physical activity ratio (PAR) value of 1.2 was used (Watkinson et al., 2010). The full list of input parameters for the Takahashi model is given in Table 2.

The two-node models (Gagge and Ji) require the vapour pressure which was calculated using Equation (11) taken from the pythermal-comfort package (Tartarini and Schiavon, 2020):

(11) $$P=\frac{RH\cdot \exp\left(18.6686-\frac{4030.183}{T_{db}+235.0}\right)}{100}$$

where, *P* is the vapour pressure (Torr); RH is the relative humidity represented in whole number form (e.g., 9% RH = 9); T_db_ is the dry bulb (air) temperature (°C).

The models also required body surface area which was calculated using the Du Bois equation taken from the pythermalcomfort package (Tartarini and Schiavon, 2020) which is represented in Equation (12):

(12) $$BSA=0.202\cdot \left(\;masss^{0.425}\right)\cdot \left(\;\text{height}s^{0.725}\right)$$

where, BSA is the body surface area (m^2^); mass of the participant (kg); and height of the participant (m).

For each model, the predicted results were compared against the experimentally recorded rectal and mean skin temperature for each participant. As the Takahashi model is a multi-node model, the pelvis segment was selected to best approximate rectal temperature, following what the authors did in their original paper (Takahashi et al., 2021).

In addition to prediction of rectal and skin temperature, we also calculated mean body temperature as per Equation (13) (Burton, 1935):

(13) $$T_{b}=0.64•T_{re}+0.36•T_{sk}$$

where *T*_*b*_ is the weighted mean body temperature (°C); *T*_*re*_ is the measured/predicted rectal temperature (°C); *T*_*sk*_ is the measured/predicted mean skin temperature (°C).

### 2.10 Error quantification

The two main metrics used for assessing model performance were Root-Mean Squared Error (RMSE) and Mean Bias Error (MBE). RMSE gives the average magnitude of error for each of the models over all minutes, while MBE gives a directional error to indicate whether a model on average under or overestimates temperature measurements. The formula for RMSE is defined in Equation (14) while the formula for MBE is given in Equation (15):

(14) $$RMSE=\sqrt{\frac{1}{n}\sum_{i=1}^{n}{\left(y_{i}-{\hat{y}}_{i}\right)}^{2}}$$

(15) $$MBE=\frac{1}{n}\sum_{i=1}^{n}\left(y_{i}-{\hat{y}}_{i}\right)$$

where, *n* is the number of data points; *y*_*i*_ is the observed value for the *i*-th data point; *ŷ*_*i*_ is the predicted value for the *i*-th data point.

To further assess the agreement between predicted and measured temperatures, limits of agreement (LoA) were calculated following Bland and Altman (1986). The LoA represent the interval within which 95% of the differences between predicted and measured values are expected to lie, and are calculated as given in Equation (16):

(16) $$LoA=MBE\pm 1.96^{*}\sigma d$$

where *MBE* is the mean bias error and *σd* is the standard deviation of the differences (yi - ŷi).

In addition to analysing overall RMSE values, we examined the proportion of participants whose individual RMSE, calculated over the entire measurement period, fell within clinically meaningful thresholds. For rectal temperature, this threshold was 0.3 °C, based on previous studies (Meade et al., 2023a; Morris et al., 2019). For mean skin temperature, the threshold was set at 1.0 °C (Bach et al., 2015; MacRae et al., 2018). This provides a measure of how well the models can predict temperatures at an individual level over the entirety of the exposure period in the temperature/humidity-controlled test chamber.

To assess the reliability of the predicted results, we calculated the 95% confidence interval (CI) for the predictions. The confidence interval gives an estimated range of values which is likely to include the true value. The formula for approximating the CI for the mean of a normally distributed variable is given by Equation (17):

(17) $$CI=\bar{x}\pm 1.96•SEM$$

where, CI is the 95% confidence interval upper and lower bounds, $\bar{x}$ is the sample mean, 1.96 is the z-value to approximate a 95% confidence interval, SEM is the standard error of the mean (Equation (18)).

(18) $$SEM=\frac{s}{\sqrt{n}}$$

where, SEM is the standard error of the mean, *s* is the standard deviation of the sample, *n* is the number of observations in the sample.

## 3 Results

The complete set of coefficients with full numerical precision for the linear and ridge regression set of models for each fold are detailed in the Supplementary material. Weights/parameters for the other models are available from our GitHub repository, along with the code used for training and validation of the models (Forbes, 2024).

**Fig. 3:**
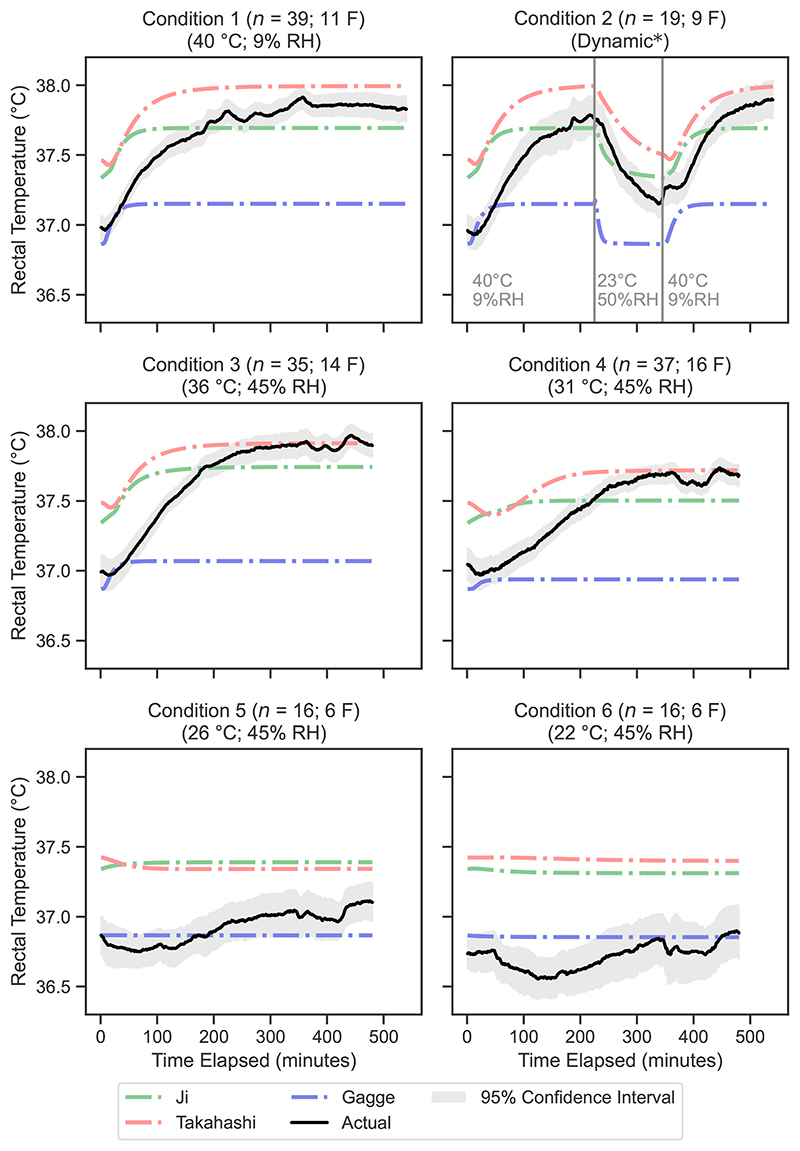
Mean predicted vs. measured rectal temperature for each minute in the validation dataset, comparing the biophysical models. F denotes the number of female participants in the validation dataset for that condition. The 95% confidence interval is calculated using Equation (17).

**Fig. 4:**
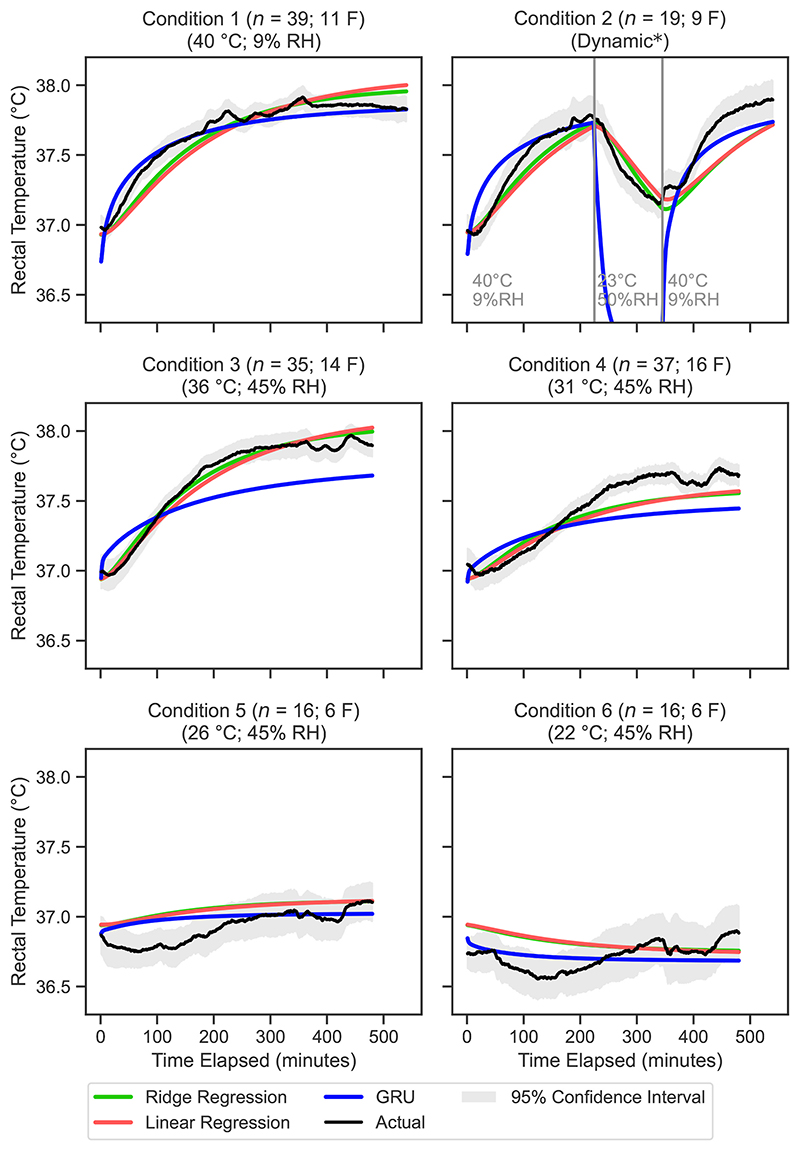
Mean predicted vs. measured rectal temperature for each minute in the validation dataset, for the top three machine learning models. The 95% confidence interval is calculated using Equation (17). RNN, recurrent neural network.

**Fig. 5:**
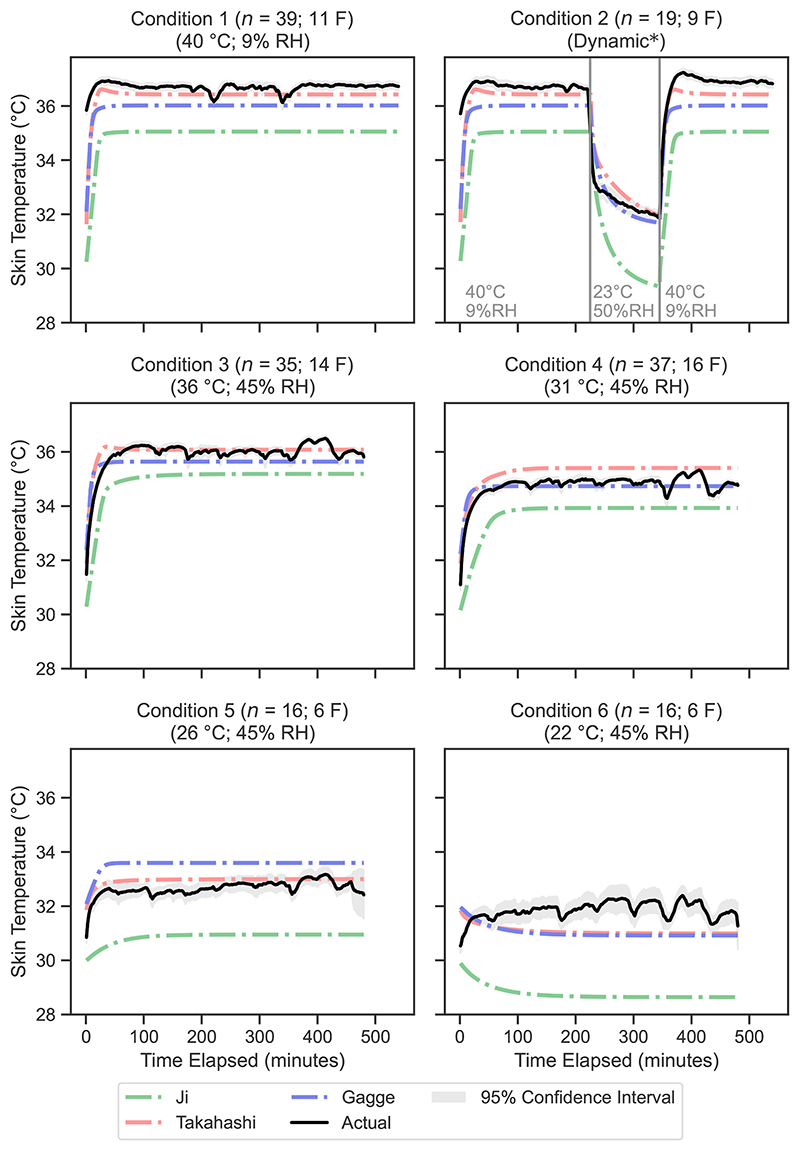
Mean predicted vs. measured skin temperature for each minute in the validation dataset, comparing the biophysical models. The 95% confidence interval is calculated using Equation (17).

**Fig. 6:**
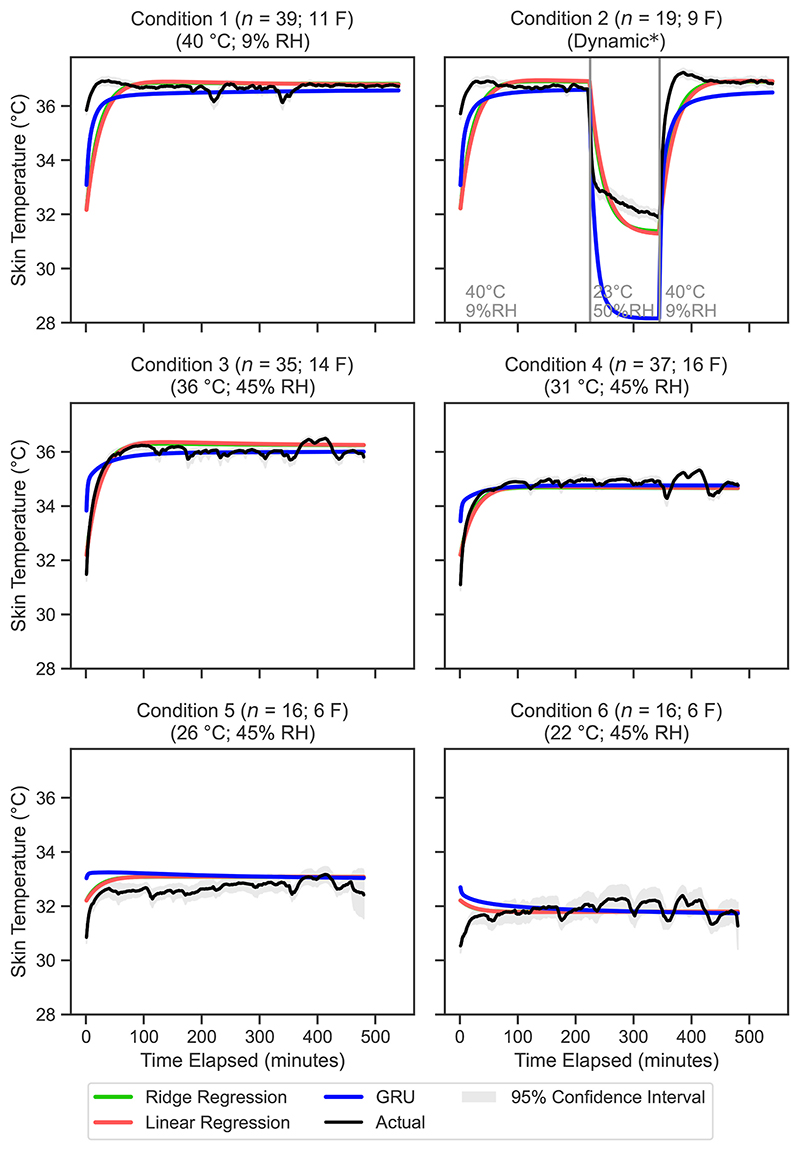
Mean predicted vs. measured skin temperature for each minute for the top three machine learning models. The 95% confidence interval is calculated using Equation (17). RNN, recurrent neural network.

The mean predicted rectal temperature values for each condition at 1-min intervals are presented in Fig. 3 for the biophysical models and in Fig. 4 for the machine learning models. Similarly, Figs. 5 and 6 display the mean predicted skin temperature values for the biophysical and machine learning models, respectively.

### 3.1 RMSE for rectal and skin temperatures

The RMSE for rectal temperature, mean skin temperature, and mean body temperature predictions over the validation dataset for each model are presented in Table 3. The ridge regression model was associated with the lowest RMSE values for all three temperature predictions, with 0.27 °C for rectal temperature, 0.73 °C for mean skin temperature, and 0.34 °C for mean body temperature. The linear regression model closely followed, with RMSE values of 0.28 °C, 0.75 °C, and 0.35 °C for rectal, skin, and body temperature, respectively.

Among the biophysical models, the Takahashi model exhibited the best performance for skin temperature and body temperature predictions (Table 3), with RMSE values of 0.74 °C and 0.39 °C, respectively. However, for rectal temperature, the Ji model outperformed the Takahashi model, with RMSE values of 0.40 °C and 0.48 °C, respectively.

The sequence prediction models, including RNN, LSTM and GRU, showed varying performance across the different temperature predictions (Fig. 4). The GRU model had the lowest RMSE values among the sequence prediction models for rectal temperature (0.35 °C), mean skin temperature (0.99 °C), and mean body temperature (0.51 °C). The RNN model performed worst out of the sequence prediction models with a RMSE of 0.43 °C for rectal temperature and 1.16 °C for skin temperature.

An analysis of RMSE values by sexes revealed no substantial differences overall (Table 4). The ridge regression model yielded rectal temperature RMSE values of 0.29 °C for males and 0.25 °C for females. For skin temperature, RMSE was 0.71 °C for males and 0.75 °C for females, while mean body temperature RMSE values were 0.35 °C for males and 0.33 °C for females. Machine learning models showed slightly higher RMSE values for males in rectal and mean body temperature predictions, while RMSE values were slightly lower for males in skin temperature predictions. Biophysical models also tended to produce higher RMSE values for males in rectal temperature predictions (with the exception of the Gagge model), however, had higher RMSE values for females in skin temperature predictions. Individual participant RMSE and MBE values for each main model are documented in Supplementary Tables S4–S9.

### 3.2 Participants within RMSE thresholds

For individual participants within RMSE thresholds, the ridge regression model had the most participants within 0.3 °C RMSE limit for rectal temperature (114 of 162 participants) and the highest within the 1.0 °C RMSE limit for skin temperature (142 of 162 participants), tied with the Takahashi model (Tables 5 and 6). Out of the biophysical models the Ji model had best performance with rectal temperature with 74 of the 162 participants within the 0.3 °C threshold.

### 3.3 MBE for rectal and skin temperatures

**Fig. 7:**
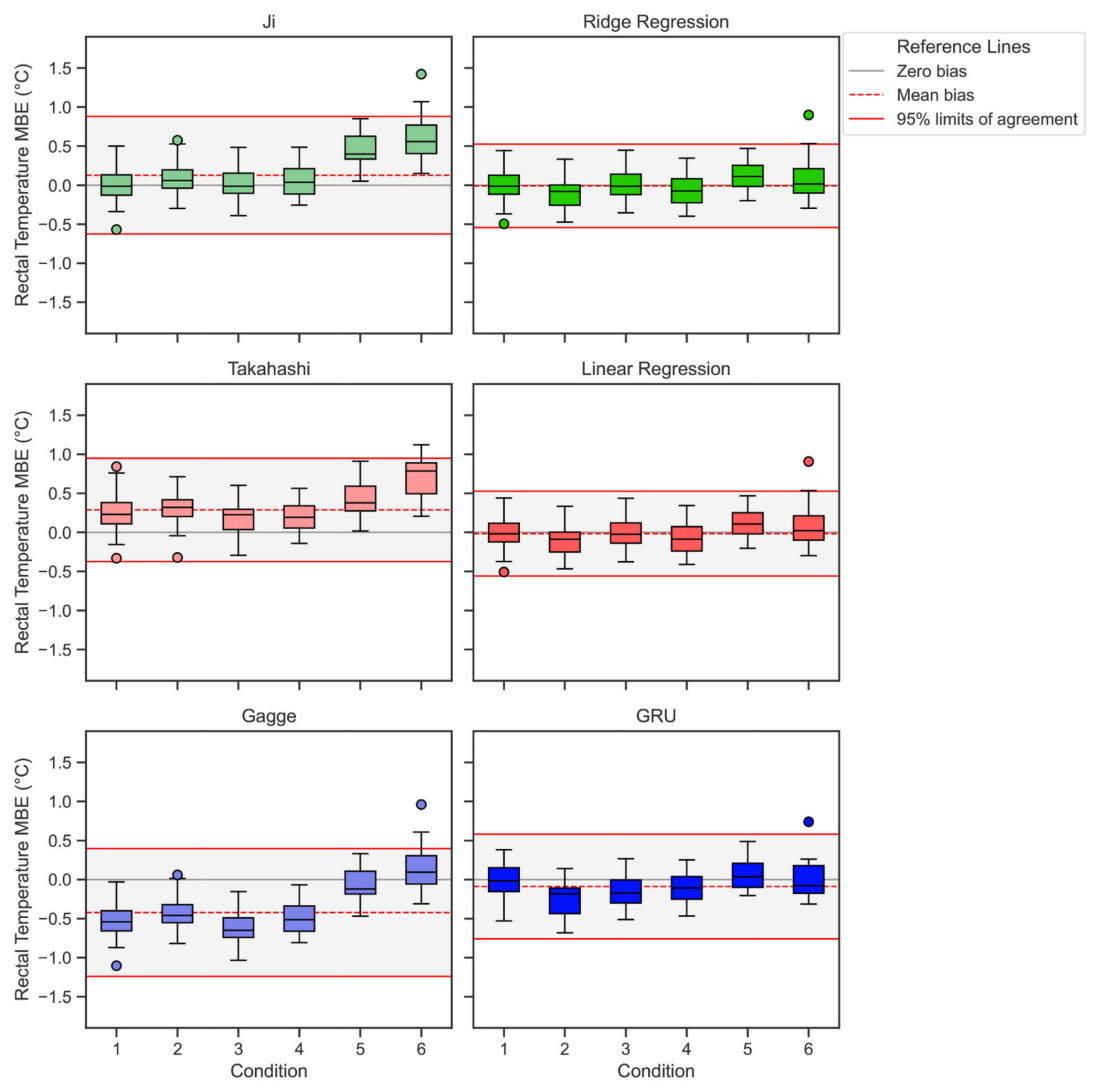
Mean Bias Error (MBE) for rectal temperature of the biophysical models vs. the top three machine learning models. Limits of agreement is calculated using Equation (16).

The MBE results for rectal temperature predictions across all six experimental conditions are presented in Fig. 7. The Gagge model consistently underestimated rectal temperature, as evidenced by negative MBE values across all conditions except for condition 6 (22 °C, 45% RH). The ridge regression, linear regression and GRU models performed similarly to each other, rarely under or overestimating rectal temperature by a large degree.

**Fig. 8:**
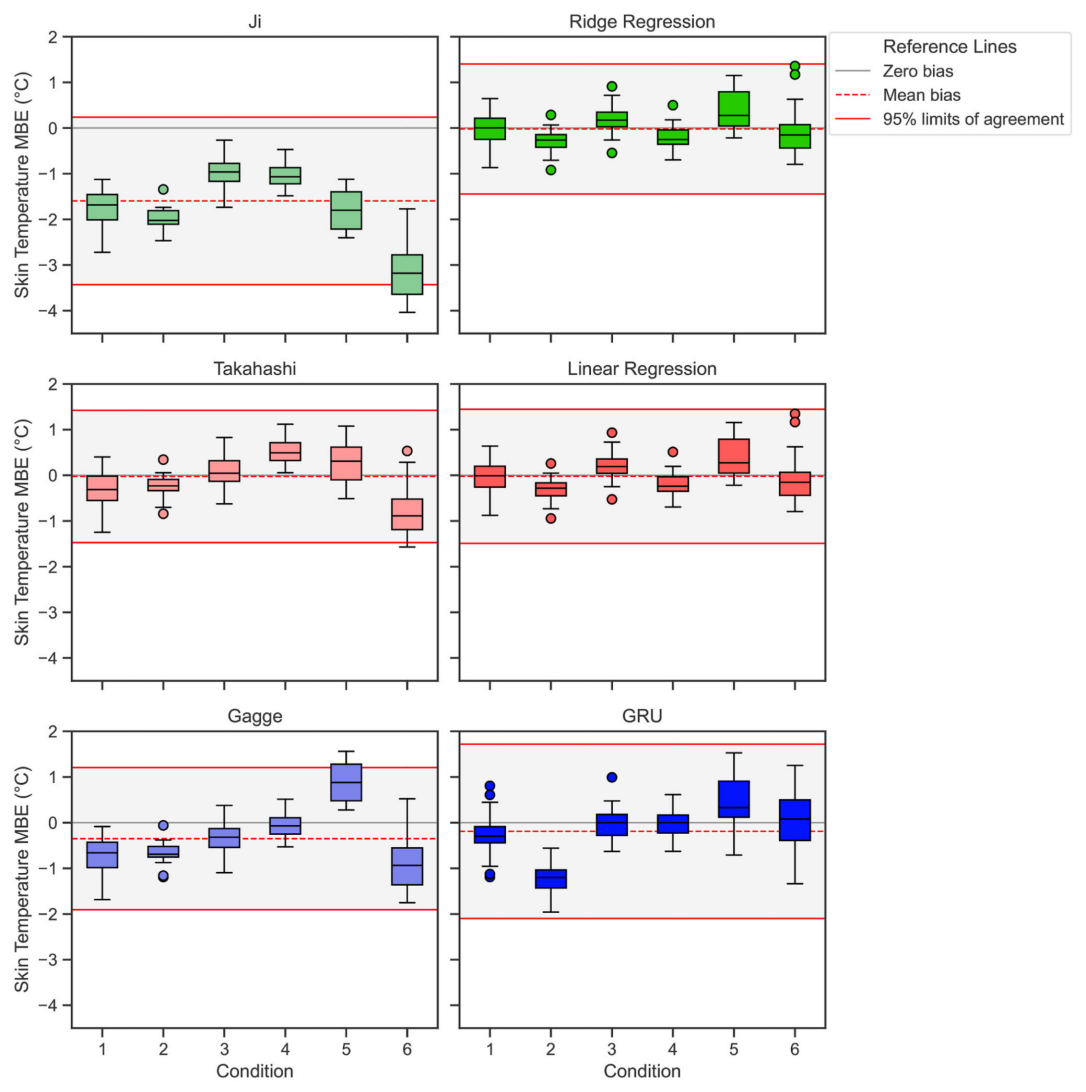
Mean bias error (MBE) for skin temperature of the biophysical models vs. the top three machine learning models. Limits of agreement is calculated using Equation (16).

The MBE for mean skin temperature predictions across all six experimental conditions are presented in Fig. 8. The Ji model consistently underestimated mean skin temperature, especially in condition 6. The Takahashi model showed mixed results, underestimating skin temperatures in conditions 1 and 6 but overestimating in condition 4 and 5. The GRU model heavily underestimates skin temperature in condition 2 but overestimates it in condition 5.

### 3.4 Sensitivity analysis

Here we investigate how participant characteristics such as age, sex, body height, body mass as well as environmental conditions (ambient temperature and relative humidity) impact the predicted rectal and skin temperature for the considered models. The model sensitivity analysis was carried out by varying one parameter at a time.

This analysis was performed with the following variables unless specified otherwise: sex = male, age = 70 years, height = 1.7 m, mass = 70 kg, ambient temperature = 35 °C, relative humidity = 45%. Conditions were simulated for 540 min, with an additional 60 min of simulation at 23 °C and 9% RH beforehand. Final predicted temperatures after the 600 min of simulation are reported in the Supplementary material.

As the Ji and Gagge models do not consider age as an input parameter, their output is the same across all age ranges. Interestingly, the ridge regression model presents a monotonic (slight increase) dependency of core temperature with age while for the RNN model the behaviour is biphasic. Takahashi’s predicted rectal temperature peaks for people aged 70 then declines (Supplement Fig. S1). This pattern occurs because physiological factors like sweating and blood flow efficiency reach their minimum values at age 70, while the basal metabolic rate continues to decrease with age according to the Harris-Benedict equation, ultimately leading to lower predicted core temperatures in advanced age. Skin temperature predictions for all models are similar to the rectal temperature predictions with a stronger positive correlation for ridge regression compared to its rectal temperature predictions (Supplement Fig. S1). The GRU model is the exception which shows a negative correlation between age and skin temperature predictions, most notably occurring in fold 1 (Supplement Fig. S2).

The Ji model provides similar predictions for both skin temperature and rectal temperature for both males and females. The Takahashi model outputs similar predictions for both male and female skin temperatures, however estimates a higher rectal temperature for males (Supplement Fig. S3). Both the ridge regression and GRU models predict higher rectal temperatures for males compared to females (Supplement Fig. S4). Ridge regression predicts higher skin temperature for males, while the GRU model tends to predict higher skin temperatures for females over all folds except for fold 4.

The Ji and Gagge models predictions for both rectal and skin temperatures are not significantly affected by height. The ridge regression, GRU and Takahashi models have a negative correlation between height and predicted skin/rectal temperatures (Supplement Figs. S5 and S6). The negative correlation between height and predicted skin/rectal temperatures observed in the GRU and ridge regression models likely arises from patterns present in the training data, where individuals with greater height may, on average, display lower temperatures. For the Takahashi model, the negative correlation with height (assuming constant weight) can be attributed to the increase in body surface area, as estimated by the Dubois equation. Increased surface area allows for more effective heat dissipation, resulting in slightly lower predicted skin/rectal temperatures.

The Takahashi model displays a positive correlation between mass and predicted skin and rectal temperatures (Supplement Fig. S7), while the Ridge Regression and GRU models generally show a negative correlation, except for rectal temperature predictions in the ridge regression model which shows a positive correlation in folds 1 and 2 (Supplement Fig. S8).

All models show a positive correlation between ambient temperature and relative humidity and predicted skin and rectal temperatures, except the GRU model which shoes slight negative correlation between relative humidity and rectal temperature, except in fold 4 (Supplement Figs. S11 and S12).

## 4 Discussion

Machine learning models, particularly ridge regression, consistently outperformed traditional biophysical models in terms of prediction accuracy, with RMSE values of 0.27 °C, 0.73 °C, and 0.34 °C for rectal,

skin, and body temperature, respectively. This aligns with our expectations, as machine learning models are built on well-defined datasets, while biophysical models attempt to provide a generalisation of the system’s dynamics. It is important to note that these biophysical models are fundamentally rational, grounded in physiological principles and thermodynamic laws. Their rational basis allows them to offer insights into the underlying mechanisms of thermoregulation, even if their predictive accuracy may be lower in specific contexts. Additionally, these models provide an advantage in terms of results interpretability due to their mechanistic foundation.

Ridge regression achieved the highest number of individual predictions within clinically meaningful RMSE bounds across most conditions, with bounds set at <0.3 °C for rectal temperature (Meade et al., 2023a; Morris et al., 2019) and <1.0 °C for skin temperature (Bach et al., 2015; MacRae et al., 2018). Specifically, 114 of 162 participants for rectal temperature and 142 of 162 participants for skin temperature fell within these bounds, as shown in Tables 5 and 6. The Takahashi model was the only model to tie with ridge regression for individual skin temperature measurements, however for rectal temperature only had 52 of the 162 participants inside the 0.3 °C limit. While the ridge regression model achieved the best results for T_re_ measurements, our findings highlight a significant limitation: approximately 30% of participants remained outside these thresholds, meaning that critical elevations in core temperature could go undetected in a substantial proportion of individuals. Given that failure to predict high core temperatures could have serious health implications, the model shows potential but requires further refinement to improve accuracy and reliability.

Linear/ridge regression most effectively capture the primary trends in rectal and skin temperatures across various heat conditions, as indicated by the lowest RMSE associated with these variables (Table 3) as well as the highest proportion of participants within clinically meaningful bounds (Tables 5 and 6) while also being a computationally inexpensive option. In addition to this, the Ridge Regression model showed good consistency over the 4-folds evaluated during cross-validation (Supplement Tables S1–3) suggesting good model generalisation for this prediction task. Individual participant analysis in Supplementary Table S7 for the ridge regression model demonstrates minimal inter-participant variation in RMSE values, with mean errors of 0.24 °C ± 0.11 °C for T_re_ and 0.74 °C ± 0.18 °C for T_sk_. While participant ID 79 exhibited the highest T_re_ RMSE of 0.51 °C, suggesting potential additional input parameters could refine individualised thermoregulatory modelling, this maximum individual RMSE remains lower than those of alternative models including the biophysical models tested here. The limited availability of comprehensive experimental data currently constrains the development of more robust machine learning models that could fully characterise diverse physiological subgroups.

Due to their simple architecture point-wise prediction models share some pitfalls. The main limitation of these models is the lack of context that they are provided with, limiting their predictive capacity where cumulative effects need to be considered (e.g., someone going from a hot environment straight into a cool environment will cool down quicker due to the sweat still on their skin). These models with their current design only ever have the previous time step in memory. Furthermore, we do not expect these models to be reliable when considering more complex environment scenarios (with variable temperature gradients), which significantly differ from the ones used for the fitting. It is also important to notice that the biophysical models were run as they were indicated by the original authors, by using a limited set of data input, without any re-parametrisation. All these aspects need to be further investigated in future research.

The point-wise prediction models (e.g., ridge and linear regression) work better in the tested datasets. However, for longer and dynamic datasets, sequence prediction models (e.g., RNN, LSTM, GRU) are expected to work better in real-world conditions as they can factor in the cumulative effects of long and repeated exposure to extreme heat. Although the tested datasets are the best available datasets we could obtain, the training data did not seem to be sufficient to train the higher parameter counts of these sequence models, which could have resulted in underfitting and higher RMSE than alternative point-wise models. Higher parameter count models have more complex structures and need more data to learn the links/relationships in the data accurately. Without enough data, these models can’t fully capture the underlying patterns and relationships in the data, resulting in poorer performance (underfitting). With more experimental data available, it is expected that sequence prediction models would perform as well if not better than the more basic regression models.

On average, the machine learning models were slightly more accurate in predicting rectal and skin temperatures for females compared to males (Table 4). Skin temperature RMSE values were similar for both sexes, but only very slightly higher for females over all machine learning models. This suggests that the lower representation of females in the training data did not adversely affect the models’ predictive accuracy for female participants.

The Ji and Takahashi biophysical models were less accurate for predicting rectal temperature in milder conditions (Fig. 3) – condition 5 (26 °C, 45% RH) and condition 6 (22 °C, 45% RH) – which increased their overall RMSE. In these conditions, the positive MBE values indicate that these models tend to overestimate rectal temperature rather than underestimate it (Fig. 7). This still means that these two models are effective as predictive tool if rectal temperature is used as a risk determinate as it is better for a model to overestimate rather than underestimate thermoregulatory strain. MBE analysis of the Gagge model however, tends to underestimate rectal temperature, which is not surprising as it was developed with a cohort of healthy younger adults in mind. This makes it unsuitable as a heat predictive model for older adults.

The ML models analysed typically exhibit neutral MBE values for both skin and rectal temperatures, indicating neither consistent under nor overestimation. However, a notable exception occurs in condition 2 (40 °C at 9% RH, then 23 °C at 50% RH, then 40 °C at 9% RH), where the GRU model significantly underestimates both skin and rectal temperatures. Examination of the temperature curves (Figs. 4 and 6) reveals that while the model fit is good for the warmer segments, the model over-estimates the drop skin and rectal temperature that occurs during the cooling period of 23 °C and 50% RH. This supports the hypothesis that there is insufficient training data in dynamic conditions for the larger sequential model types.

The development of more accurate core and skin temperatures forecasting methods, as demonstrated in this study, has significant potential to enhance real-time predictive heat-health monitoring, particularly for vulnerable populations such as older adults (Kenny et al., 2010). Typically large scale early warning systems for heat-related risks are based on health outcomes like mortality and morbidity rather than physiological measures alone (Issa et al., 2021). However, these models are usually built upon population level epidemiological data with lack of individualisation for personal characteristics and parameters (such as height, weight, health status, etc.). By providing more precise, individualised predictions, these models can help bridge the gap between population-level data and personal risk factors. This improved accuracy could lead to more targeted and timely interventions, potentially reducing heat-related morbidity and mortality. For instance, healthcare providers and caregivers could use these predictions to implement preventative and/or mitigation measures before an individual reaches dangerous core temperature levels (Deng et al., 2018; Ou et al., 2023; Zhao et al., 2020). Additionally, safe exposure times could be estimated before heat mitigation actions need to be implemented (such as turning on air conditioner or transfer to a cooler room). Moreover, these models could be integrated into smart home systems or wearable devices, offering real-time, personalised heat risk assessment and guidance (Son et al., 2021). Future research should focus on validating these models in real-world settings, incorporating a wider range of environmental conditions and individual factors. Expansion into other promising model types such as physics-informed neural networks could also be investigated in future research (Raissi et al., 2019). Additionally, interdisciplinary collaboration between data scientists, physiologists, and public health experts will be crucial to translate these improved prediction methods into practical, user-friendly early and/or real-time warning systems (Oberai et al., 2025). The goal is to create a more responsive and effective heat management strategies that can adapt to the increasing challenges posed by climate change and urbanisation.

### 4.1 Limitations

Several limitations must be acknowledged in this study. Firstly, the linear and ridge regression models may be especially prone to error propagation over longer time durations due to their point-wise prediction nature. While the data fits well over the longest time frame investigated here (540 min, Figs. 4 and 6), extending over longer time frames typical of multi-day heat event for example, may not be valid and investigation on even longer time series data is recommended. Furthermore, the data is taken from studies which maintain exposure at a fixed condition, where in real-world applications the indoor temperature will gradually increase over the day and decrease overnight based on outdoor temperature, which can also vary across days of exposure in multi-day heat events (Kenny et al., 2024). Ideally, future datasets will be sourced from multi-day, in home trials to closer align data to what is expected under real-world conditions.

Secondly, the sequence-based models, such as GRU, LSTM, and RNN, are trained on specific sequence lengths, which limits how much context (information) they can retain in their memory. As a result, these models might need further refinement to improve their long-term prediction capabilities. Furthermore, the exploration of the model architecture and hyperparameter space was constrained by computational resources. For instance, only a limited number of unit sizes for the recurrent layers were evaluated, and other architectural choices, such as the number of layers or the use of different regularisation techniques, were not extensively investigated. This may have resulted in a suboptimal model configuration. Additionally, sequential machine learning models, like those employed in this study, can be more sensitive to variations in input data compared to simpler models such as linear or ridge regression. This ‘brittleness’ means that their performance may degrade when exposed to unseen data that deviates significantly from the training distribution, potentially limiting their generalisability in real-world scenarios.

Thirdly, the models were trained on a relatively small dataset (by machine learning standards) in mostly static conditions (excluding Condition 2). More dynamic conditions or static conditions that fall outside of the tested ranges requires validation. However, it is important to note that the dataset used in this study is of high quality relative to other heat exposure studies, with many individual exposures (162) over a long duration of exposure (480–540 min).

Lastly, the current models do not include other meaningful environmental factors as inputs such as: air velocity (Meade et al., 2024b), radiative heat transfer (Turner et al., 2023), clothing level (Gavin, 2003), and an individual’s activity level (Gleeson, 1998). This is due to there being little variation of these parameters in the dataset, therefore the machine learning models cannot learn how variation of these parameters affects body temperature. The omission of these parameters in the current machine learning models limits their real-world applicability in their current form and is an area for further research.

## 5 Conclusion

This study demonstrates that tailored machine learning models, particularly ridge regression, outperformed traditional biophysical models in predicting individualised thermoregulatory responses within the specific datasets tested. Ridge regression showed the lowest RMSE for T_re_, T_sk_ and body temperature measurements (0.27, 0.73 and 0.34 °C respectively) while also having the highest number of participant RMSEs within clinically meaningful thresholds for both T_re_ (<0.3 °C, 70%) and T_sk_ (<1.0 °C, 88%). However, the models’ performance in dynamic conditions and in long-term predictions remains to be further understood. Despite these constraints, the increasing availability of biophysical data from wearable sensors and advanced measurement methods highlights the potential for machine learning in creating accurate, individualised thermoregulatory predictions. Future research should focus on larger training datasets where possible, incorporate more dynamic conditions, activities and environmental factors, and validate models in diverse real-world scenarios. Refining these models could significantly enhance personalised heat management strategies, particularly for vulnerable populations.

## Supplementary Material

Supplementary data to this article can be found online at https://doi.org/10.1016/j.jtherbio.2025.104078.

Supplementary Material

## Figures and Tables

**Table 1:** Validation participant split between different conditions.

Condition	Fold 1	Fold 2	Fold 3	Fold 4
1	9	11	9	10
2	3	5	5	6
3	10	10	8	7
4	11	10	9	7
5	4	4	4	4
6	4	4	4	4
**Total**	**41**	**44**	**39**	**38**

*Note:* Some participants were included in multiple conditions. The training/validation set was stratified based on unique individuals, with 40% of unique individuals assigned to the training group.

**Table 2:** Input parameters used with the biophysical models.

Input variable	Value
**All biophysical models (Gagge, Ji, Takahashi)**	
Clothing value (clo)	0.23 (except for males in condition 1 and 2 where clo = 0.15)
Air velocity	0.1 m s^–1^
**Two-node models (Gagge, Ji)**	
Dry bulb temperature (T_db_)	Same as ambient temperature (°C)
Radiant temperature (T_r_)	Same as ambient temperature (°C)
Metabolic rate (MET)	1.3 kcal kg^–1^-h^–1^
Vapour pressure	Calculated using Equation 11
Atmospheric pressure	101,325 Pa
Body position	Sitting
Body surface area (BSA)	Calculated using Equation 12
**Optimised two-node model (Ji)**	
Body mass	Measured mass (kg)
Heat acclimatised	Yes
**JOS-3 model (Takahashi)**	
Body height	Measured height (m)
Body mass	Measured mass (kg)
Body fat	Measured body fat percentage
Sex	Indicated by participant
Physical activity ratio (PAR)	1.2
Ambient temperature (T_a_)	Same as ambient temperature of test chamber (°C)
Radiant temperature (T_r_)	Same as ambient temperature (°C)
Relative humidity (RH)	Same as relative humidity of test chamber (% RH)

**Table 3:** RMSE (°C) over the validation dataset for each model.

Model	Rectal	Skin	Mean Body
**Biophysical**			
Ji	0.40	1.85	0.62
Takahashi	0.44	0.74	0.39
Gagge	0.59	0.87	0.58
**Machine Learning**			
RidgeR	0.27	0.73	0.34
LinearR	0.28	0.75	0.35
GRU	0.35	0.99	0.51
LSTM	0.42	1.12	0.57
RNN	0.43	1.16	0.60

*Note:* RMSE, root mean square error; RidgeR, ridge regression; LinearR, linear regression; RNN, recurrent neural network; LSTM, long-short term memory networks; GRU, gated recurrent units.

**Table 4:** RMSE (°C) over the validation dataset for each model, comparing male and female results.

Model	Rectal Male	Rectal Female	Skin Male	Skin Female	Mean Body Male	Mean Body Female
**Biophysical**						
Ji	0.44	0.33	1.77	1.97	0.56	0.70
Takahashi	0.48	0.38	0.67	0.84	0.40	0.37
Gagge	0.56	0.64	0.79	0.98	0.54	0.64
**Machine Learning**						
RidgeR	0.29	0.25	0.71	0.75	0.35	0.33
LinearR	0.29	0.25	0.74	0.77	0.36	0.34
GRU	0.36	0.35	0.99	1.00	0.51	0.50
LSTM	0.42	0.41	1.09	1.15	0.58	0.55
RNN	0.44	0.41	1.15	1.19	0.61	0.57

*Note:* RMSE, root mean square error; RidgeR, ridge regression; LinearR, linear regression; RNN, recurrent neural network; LSTM, long-short term memory networks; GRU, gated recurrent units.

**Table 5:** Fraction of participant exposures (*n* = 162) within clinically meaningful rectal temperature limits (0.3 °C RMSE).

Condition	Biophysical				Machine learning				
	Ji	Takahashi	Gagge		RidgeR	LinearR	GRU	LSTM	RNN
1	24/39	18/39	3/39		30/39	29/39	28/39	17/39	15/39
2	9/19	3/19	3/19		12/19	12/19	0/19	8/19	7/19
3	19/35	16/35	0/35		26/35	25/35	18/35	6/35	5/35
4	18/37	17/37	2/37		26/37	26/37	22/37	8/37	8/37
5	2/16	2/16	10/16		10/16	10/16	11/16	8/16	8/16
6	2/16	0/16	9/16		10/16	10/16	11/16	6/16	7/16
**Overall**	**74/162**	**56/162**	**27/162**		**114/162**	**112/162**	**90/162**	**53/162**	**50/162**

*Note:* RMSE, root mean square error; RidgeR, ridge regression; LinearR, linear regression; RNN, recurrent neural network; LSTM, long-short term memory networks; GRU, gated recurrent units.

**Table 6:** Fraction of participants (*n* = 66) within clinically meaningful skin temperature limits (1.0 °C RMSE).

Condition	Biophysical				Machine learning				
	Ji	Takahashi	Gagge		RidgeR	LinearR	GRU	LSTM	RNN
1	0/39	36/39	25/39		37/39	35/39	34/39	28/39	23/39
2	0/19	18/19	14/19		11/19	8/19	0/19	4/19	4/19
3	12/35	35/35	33/35		34/35	34/35	33/35	18/35	19/35
4	7/37	33/37	37/37		37/37	37/37	36/37	32/37	31/37
5	0/16	14/16	8/16		11/16	11/16	10/16	5/16	6/16
6	0/16	6/16	7/16		12/16	12/16	13/16	2/16	1/16
**Overall**	**19/162**	**142/162**	**124/162**		**142/162**	**137/162**	**126/162**	**89/162**	**84/162**

*Note:* RMSE, root mean square error; RidgeR, ridge regression; LinearR, linear regression; RNN, recurrent neural network; LSTM, long-short term memory networks; GRU, gated recurrent units

## Data Availability

Weights/parameters for the models in this manuscript are available from our GitHub repository, along with the code used for training and validation of the models (https://github.com/climate-ethos/core-temperature-modelling). The deidentified participant data used to train the models are available by contacting Glen Kenny (gkenny@uottawa.ca) upon reasonable request and signed access agreement.

## Data availability

No
