# Supplementary material for: Biophysical versus machine learning models for predicting rectal and skin temperatures in older adults
Source: J Therm Biol. Author manuscript; Available in PMC 2026 Jan 6. (PMC7618561; doi:10.1016/j.jtherbio.2025.104078)
Supplement: Supplementary Material [file EMS211702-supplement-Supplementary_Material.pdf]

Supplementary Material

1 Linear Regression Coefficients and Intercepts

The linear regression coefficients with full numerical precision for rectal temperature prediction (y_{tre}) and skin temperature prediction (y_{mtsk}) are presented in the below sections. Where: Ta = ambient temperature (°C), RH = relative humidity (%), Tre = rectal temperature (°C), Tsk = skin temperature (°C).

1.1 Fold 1

1.1.1 Rectal Temperature

$$\beta_1(\text{sex}) = 1.1609365600120396 \times 10^{-4}$$
$$\beta_2(\text{age}) = 6.9601415042941372 \times 10^{-4}$$
$$\beta_3(\text{height}) = -2.9525954572105704 \times 10^{-4}$$
$$\beta_4(\text{mass}) = 3.1591690770997989 \times 10^{-4}$$
$$\beta_5(\text{Ta}) = 7.4772726100831512 \times 10^{-4}$$
$$\beta_6(\text{RH}) = 5.6225044657834191 \times 10^{-4}$$
$$\beta_7(\text{previous Tre}) = 9.9453001653721476 \times 10^{-1}$$
$$\beta_8(\text{previous Tsk}) = 5.2246659119323287 \times 10^{-3}$$
$$\beta_c = -1.4011488914054127 \times 10^{-3}$$

1.1.2 Skin Temperature

$$\beta_1'(\text{sex}) = 5.8916401630318243 \times 10^{-4}$$
$$\beta_2'(\text{age}) = 2.0111202785891291 \times 10^{-4}$$
$$\beta_3'(\text{height}) = -4.5672013032092966 \times 10^{-4}$$
$$\beta_4'(\text{mass}) = -9.1125317907826489 \times 10^{-4}$$
$$\beta_5'(\text{Ta}) = 1.6961667329947836 \times 10^{-2}$$
$$\beta_6'(\text{RH}) = 3.0034091385099924 \times 10^{-3}$$
$$\beta_7'(\text{previous Tre}) = -1.8242320489997710 \times 10^{-3}$$
$$\beta_8'(\text{previous Tsk}) = 9.3893935457056248 \times 10^{-1}$$
$$\beta_c' = 4.1569797121632668 \times 10^{-2}$$

1.2 Fold 2

1.2.1 Rectal Temperature

$$\beta_1(\text{sex}) = 9.1625302291432149 \times 10^{-5}$$
$$\beta_2(\text{age}) = 6.4793987521641343 \times 10^{-4}$$
$$\beta_3(\text{height}) = -6.1336258558948460 \times 10^{-5}$$
$$\beta_4(\text{mass}) = 2.7749507145394205 \times 10^{-4}$$
$$\beta_5(\text{Ta}) = 8.5524543737973158 \times 10^{-4}$$
$$\beta_6(\text{RH}) = 4.9136672704181654 \times 10^{-4}$$
$$\beta_7(\text{previous Tre}) = 9.9547553502779884 \times 10^{-1}$$
$$\beta_8(\text{previous Tsk}) = 4.1585556864743607 \times 10^{-3}$$
$$\beta_c = -1.3025823168477668 \times 10^{-3}$$

1.2.2 Skin Temperature

$$\beta_1'(\text{sex}) = 4.7916153951258771 \times 10^{-4}$$
$$\beta_2'(\text{age}) = 4.6981325648624841 \times 10^{-4}$$
$$\beta_3'(\text{height}) = -4.3221280447744963 \times 10^{-4}$$
$$\beta_4'(\text{mass}) = -5.8621994237220614 \times 10^{-4}$$
$$\beta_5'(\text{Ta}) = 1.5638608555418692 \times 10^{-2}$$
$$\beta_6'(\text{RH}) = 2.4863174900283904 \times 10^{-3}$$
$$\beta_7'(\text{previous Tre}) = -1.7662149511905727 \times 10^{-3}$$
$$\beta_8'(\text{previous Tsk}) = 9.4123926168426646 \times 10^{-1}$$
$$\beta_c' = 4.0757333122858785 \times 10^{-2}$$

1.3 Fold 3

1.3.1 Rectal Temperature

$$\beta_1(\text{sex}) = 1.3418818910516213 \times 10^{-4}$$
$$\beta_2(\text{age}) = 7.5322683173317806 \times 10^{-4}$$
$$\beta_3(\text{height}) = -2.5980070739598773 \times 10^{-4}$$
$$\beta_4(\text{mass}) = -7.0371852897125642 \times 10^{-5}$$
$$\beta_5(\text{Ta}) = 1.0175898302452633 \times 10^{-3}$$
$$\beta_6(\text{RH}) = 5.6075318958053266 \times 10^{-4}$$
$$\beta_7(\text{previous Tre}) = 9.9473389506982031 \times 10^{-1}$$
$$\beta_8(\text{previous Tsk}) = 4.4181546669846741 \times 10^{-3}$$
$$\beta_c = -1.0255387825283124 \times 10^{-3}$$

1.3.2 Skin Temperature

$$\beta_1'(\text{sex}) = 4.8589344530694873 \times 10^{-4}$$
$$\beta_2'(\text{age}) = 4.9469086983538331 \times 10^{-4}$$
$$\beta_3'(\text{height}) = -1.9745892386332000 \times 10^{-4}$$
$$\beta_4'(\text{mass}) = -1.5259512013711658 \times 10^{-3}$$
$$\beta_5'(\text{Ta}) = 1.6428037316771567 \times 10^{-2}$$
$$\beta_6'(\text{RH}) = 2.4615453533733334 \times 10^{-3}$$
$$\beta_7'(\text{previous Tre}) = -1.7959628079831240 \times 10^{-3}$$
$$\beta_8'(\text{previous Tsk}) = 9.3936338418640197 \times 10^{-1}$$
$$\beta_c' = 4.1804311445859836 \times 10^{-2}$$

1.4 Fold 4

1.4.1 Rectal Temperature

$$\beta_1(\text{sex}) = 1.5416270694002092 \times 10^{-4}$$
$$\beta_2(\text{age}) = 7.1385409313645676 \times 10^{-4}$$
$$\beta_3(\text{height}) = 5.5819895959553048 \times 10^{-5}$$
$$\beta_4(\text{mass}) = 7.3136976157161318 \times 10^{-5}$$
$$\beta_5(\text{Ta}) = 6.9179197476575203 \times 10^{-4}$$
$$\beta_6(\text{RH}) = 4.8694088172136425 \times 10^{-4}$$
$$\beta_7(\text{previous Tre}) = 9.9485756065489479 \times 10^{-1}$$
$$\beta_8(\text{previous Tsk}) = 4.8003302209875134 \times 10^{-3}$$
$$\beta_c = -1.3085726136563558 \times 10^{-3}$$

1.4.2 Skin Temperature

$$\beta_1'(\text{sex}) = 4.8749852038015336 \times 10^{-4}$$
$$\beta_2'(\text{age}) = 5.0004171217553353 \times 10^{-4}$$
$$\beta_3'(\text{height}) = -2.1347037362161485 \times 10^{-4}$$
$$\beta_4'(\text{mass}) = -1.6640365276984210 \times 10^{-3}$$
$$\beta_5'(\text{Ta}) = 1.8724245770493100 \times 10^{-2}$$
$$\beta_6'(\text{RH}) = 2.7995727051080346 \times 10^{-3}$$
$$\beta_7'(\text{previous Tre}) = -1.9747545175299921 \times 10^{-3}$$
$$\beta_8'(\text{previous Tsk}) = 9.3445732382606828 \times 10^{-1}$$
$$\beta_c' = 4.4063507817612813 \times 10^{-2}$$

2 Ridge Regression Coefficients and Intercepts

2.1 Fold 1

2.1.1 Rectal Temperature

$$\beta_1(\text{sex}) = 1.6261586852849348 \times 10^{-4}$$
$$\beta_2(\text{age}) = 7.3681421437795942 \times 10^{-4}$$
$$\beta_3(\text{height}) = -4.3916987857211636 \times 10^{-4}$$
$$\beta_4(\text{mass}) = 4.6532701146677997 \times 10^{-4}$$
$$\beta_5(\text{Ta}) = 8.4439348066203674 \times 10^{-4}$$
$$\beta_6(\text{RH}) = 6.6633790662377139 \times 10^{-4}$$
$$\beta_7(\text{previous Tre}) = 9.9328104284890557 \times 10^{-1}$$
$$\beta_8(\text{previous Tsk}) = 6.0162332082507906 \times 10^{-3}$$
$$\beta_c = -1.3528489525256315 \times 10^{-3}$$

2.1.2 Skin Temperature

$$\beta_1'(\text{sex}) = 6.1578454528691511 \times 10^{-4}$$
$$\beta_2'(\text{age}) = 1.4854705372386214 \times 10^{-4}$$
$$\beta_3'(\text{height}) = -4.3298261693481379 \times 10^{-4}$$
$$\beta_4'(\text{mass}) = -1.1471088118388912 \times 10^{-3}$$
$$\beta_5'(\text{Ta}) = 1.8904677058503336 \times 10^{-2}$$
$$\beta_6'(\text{RH}) = 3.1889957127636559 \times 10^{-3}$$
$$\beta_7'(\text{previous Tre}) = -1.0477636196332154 \times 10^{-3}$$
$$\beta_8'(\text{previous Tsk}) = 9.3391821058056301 \times 10^{-1}$$
$$\beta_c' = 4.3563287283298395 \times 10^{-2}$$

2.2 Fold 2

2.2.1 Rectal Temperature

$$\beta_1(\text{sex}) = 1.3267726822439354 \times 10^{-4}$$
$$\beta_2(\text{age}) = 6.9679323387469188 \times 10^{-4}$$
$$\beta_3(\text{height}) = -1.4481042835498017 \times 10^{-4}$$
$$\beta_4(\text{mass}) = 4.2251491903380325 \times 10^{-4}$$
$$\beta_5(\text{Ta}) = 9.2909701343924365 \times 10^{-4}$$
$$\beta_6(\text{RH}) = 5.8593648556208260 \times 10^{-4}$$
$$\beta_7(\text{previous Tre}) = 9.9418293927202779 \times 10^{-1}$$
$$\beta_8(\text{previous Tsk}) = 5.0470706307470676 \times 10^{-3}$$
$$\beta_c = -1.3172254636907299 \times 10^{-3}$$

2.2.2 Skin Temperature

$$\beta'_1(\text{sex}) = 4.9351735970996655 \times 10^{-4}$$
$$\beta'_2(\text{age}) = 4.4234635301075187 \times 10^{-4}$$
$$\beta'_3(\text{height}) = -4.0806374585494858 \times 10^{-4}$$
$$\beta'_4(\text{mass}) = -8.0773822050904567 \times 10^{-4}$$
$$\beta'_5(\text{Ta}) = 1.7540705798834808 \times 10^{-2}$$
$$\beta'_6(\text{RH}) = 2.6214533408908682 \times 10^{-3}$$
$$\beta'_7(\text{previous Tre}) = -9.0279994476532899 \times 10^{-4}$$
$$\beta'_8(\text{previous Tsk}) = 9.3615328007414433 \times 10^{-1}$$
$$\beta'_c = 4.2794299156249105 \times 10^{-2}$$

2.3 Fold 3

2.3.1 Rectal Temperature

$$\beta_1(\text{sex}) = 1.6353871958580193 \times 10^{-4}$$
$$\beta_2(\text{age}) = 8.1286305542003401 \times 10^{-4}$$
$$\beta_3(\text{height}) = -3.7960078665114265 \times 10^{-4}$$
$$\beta_4(\text{mass}) = -2.5276689478970851 \times 10^{-5}$$
$$\beta_5(\text{Ta}) = 1.1145153643878023 \times 10^{-3}$$
$$\beta_6(\text{RH}) = 6.5636174911195191 \times 10^{-4}$$
$$\beta_7(\text{previous Tre}) = 9.9352048219778943 \times 10^{-1}$$
$$\beta_8(\text{previous Tsk}) = 5.1830986557812073 \times 10^{-3}$$
$$\beta_c = -9.5491223215848908 \times 10^{-4}$$

2.3.2 Skin Temperature

$$\beta'_1(\text{sex}) = 5.0011959518728091 \times 10^{-4}$$
$$\beta'_2(\text{age}) = 4.6371288690013461 \times 10^{-4}$$
$$\beta'_3(\text{height}) = -1.2614491712820057 \times 10^{-4}$$
$$\beta'_4(\text{mass}) = -1.7666966253587746 \times 10^{-3}$$
$$\beta'_5(\text{Ta}) = 1.8211263043274406 \times 10^{-2}$$
$$\beta'_6(\text{RH}) = 2.5762100711064049 \times 10^{-3}$$
$$\beta'_7(\text{previous Tre}) = -1.0504506878065885 \times 10^{-3}$$
$$\beta'_8(\text{previous Tsk}) = 9.3463829132428735 \times 10^{-1}$$
$$\beta'_c = 4.3704300584683109 \times 10^{-2}$$

2.4 Fold 4

2.4.1 Rectal Temperature

$$\beta_1(\text{sex}) = 1.9016048939670118 \times 10^{-4}$$
$$\beta_2(\text{age}) = 7.6683157269690874 \times 10^{-4}$$
$$\beta_3(\text{height}) = -2.2664846633118478 \times 10^{-6}$$
$$\beta_4(\text{mass}) = 1.2713601866032502 \times 10^{-4}$$
$$\beta_5(\text{Ta}) = 8.2295707482645462 \times 10^{-4}$$
$$\beta_6(\text{RH}) = 5.9918088253635329 \times 10^{-4}$$
$$\beta_7(\text{previous Tre}) = 9.9358708052995617 \times 10^{-1}$$
$$\beta_8(\text{previous Tsk}) = 5.5288644792845671 \times 10^{-3}$$
$$\beta_c = -1.2358382975200533 \times 10^{-3}$$

2.4.2 Skin Temperature

$$\beta'_1(\text{sex}) = 5.0470176227044163 \times 10^{-4}$$
$$\beta'_2(\text{age}) = 4.7654832837464900 \times 10^{-4}$$
$$\beta'_3(\text{height}) = -2.0442902203981319 \times 10^{-4}$$
$$\beta'_4(\text{mass}) = -1.8890177875050009 \times 10^{-3}$$
$$\beta'_5(\text{Ta}) = 2.0657387461247616 \times 10^{-2}$$
$$\beta'_6(\text{RH}) = 2.9300973743995633 \times 10^{-3}$$
$$\beta'_7(\text{previous Tre}) = -1.2624016228437533 \times 10^{-3}$$
$$\beta'_8(\text{previous Tsk}) = 9.2945128283434748 \times 10^{-1}$$
$$\beta'_c = 4.6114957482371048 \times 10^{-2}$$

3 Sensitivity Analysis

This analysis was performed with the following variables unless specified otherwise: sex = male, age = 70, height = 1.7 m, mass = 70 kg, ambient temperature = 35°C, relative humidity = 45%. Conditions were simulated for 540 minutes, with an additional 60 minutes of simulation at 23 and 9% RH beforehand. Final predicted temperatures after the 600 minutes of simulation are reported in the Figures.

3.1 Age

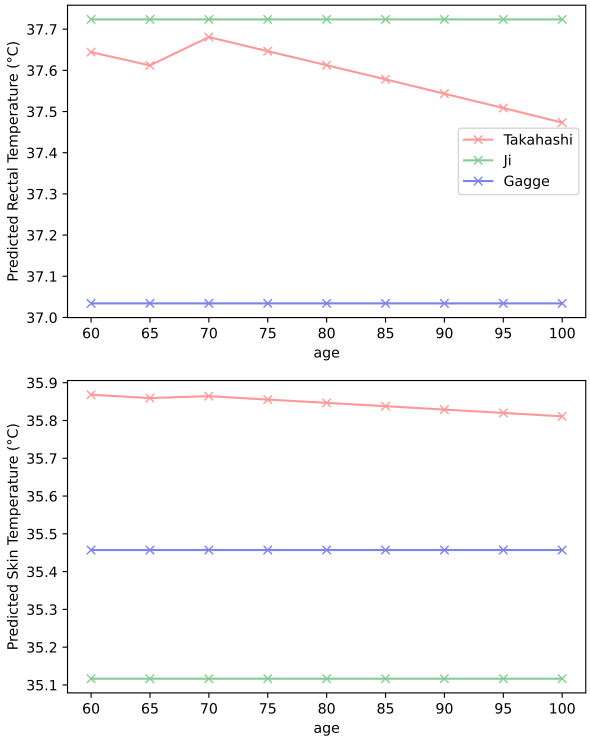

Figure S1: Predicted rectal and skin temperature over various ages for the biophysical models

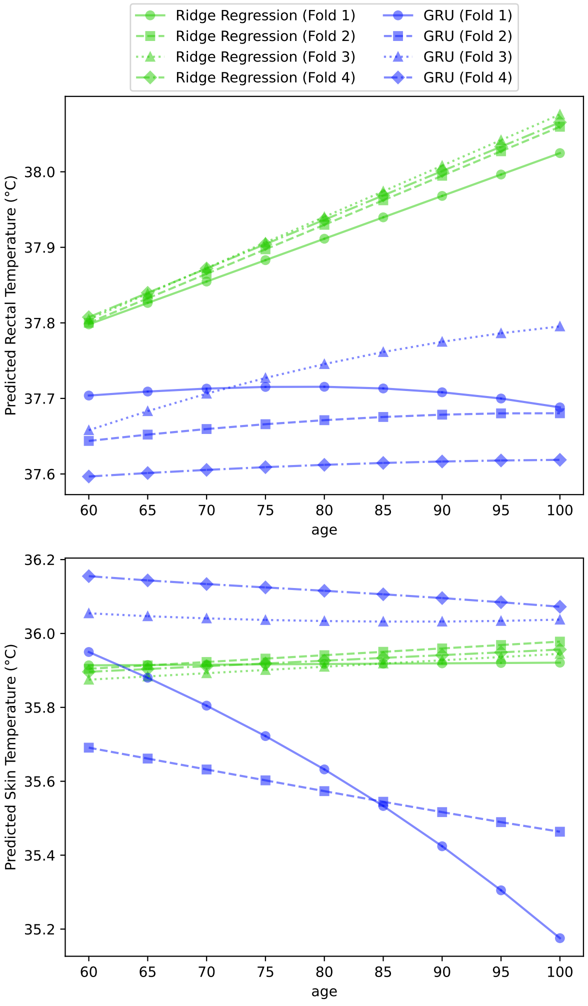

Figure S2: Predicted rectal and skin temperature over various ages for the ML models

3.2 Sex

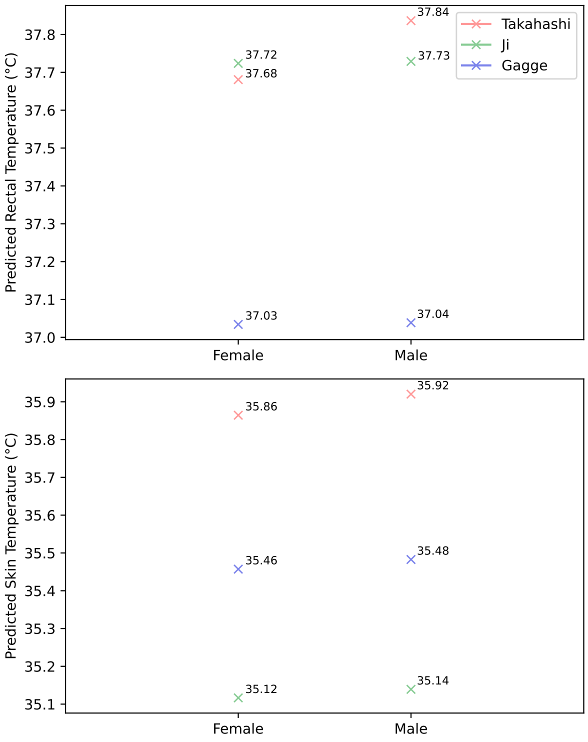

Figure S3: Predicted rectal and skin temperature for male (0) and female (1) participants for the biophysical models

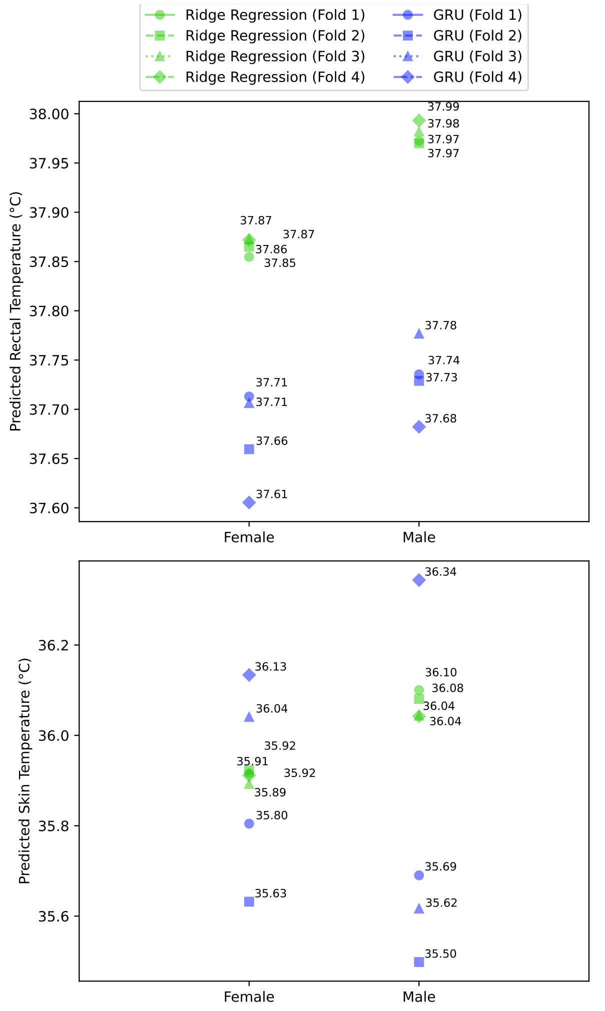

Figure S4: Predicted rectal and skin temperature for male (0) and female (1) participants for the ML models

3.3 Height

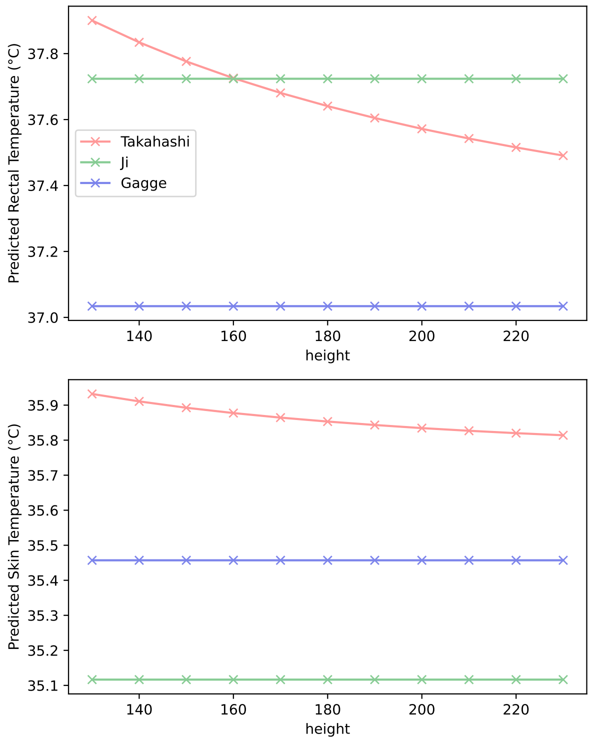

Figure S5: Predicted rectal and skin temperature over various heights for the biophysical models

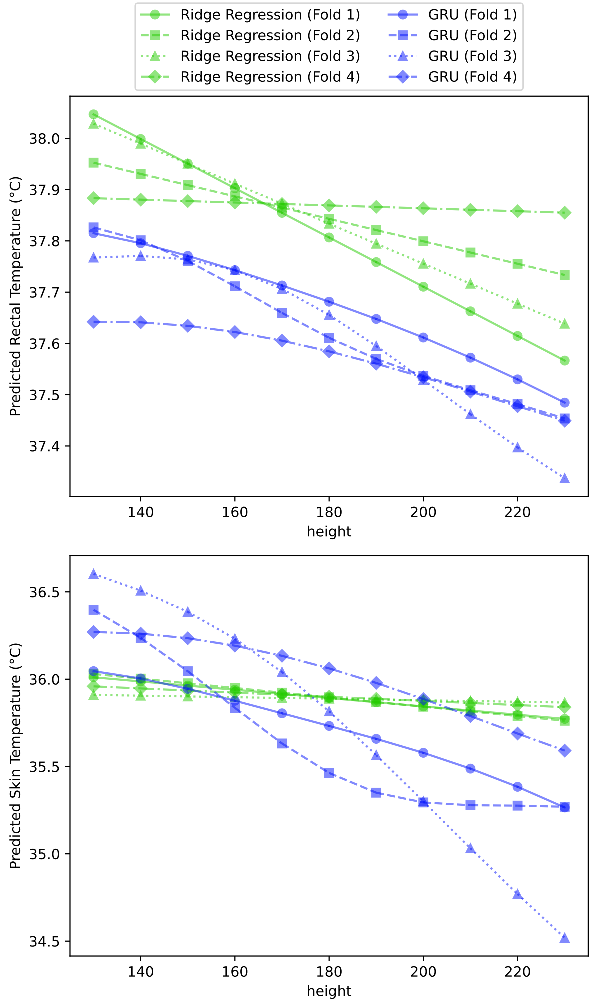

Figure S6: Predicted rectal and skin temperature over various heights for the ML models

3.4 Mass

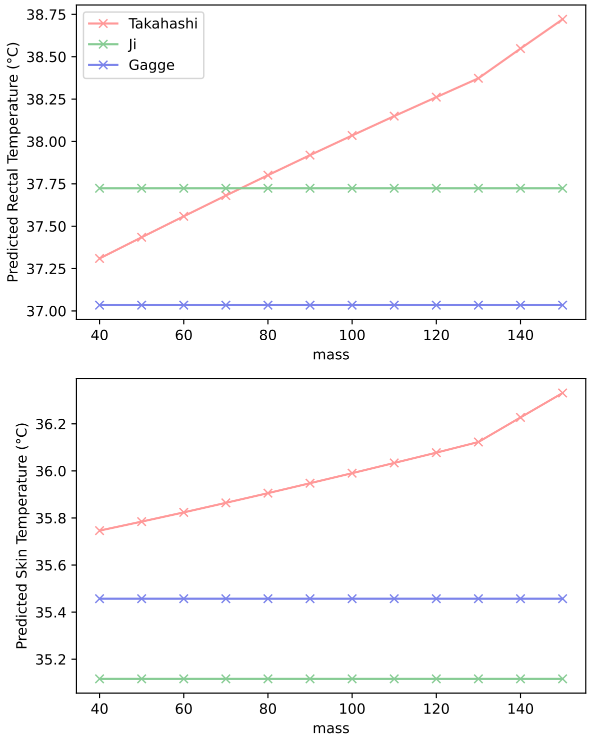

Figure S7: Predicted rectal and skin temperature over various body masses for the biophysical models

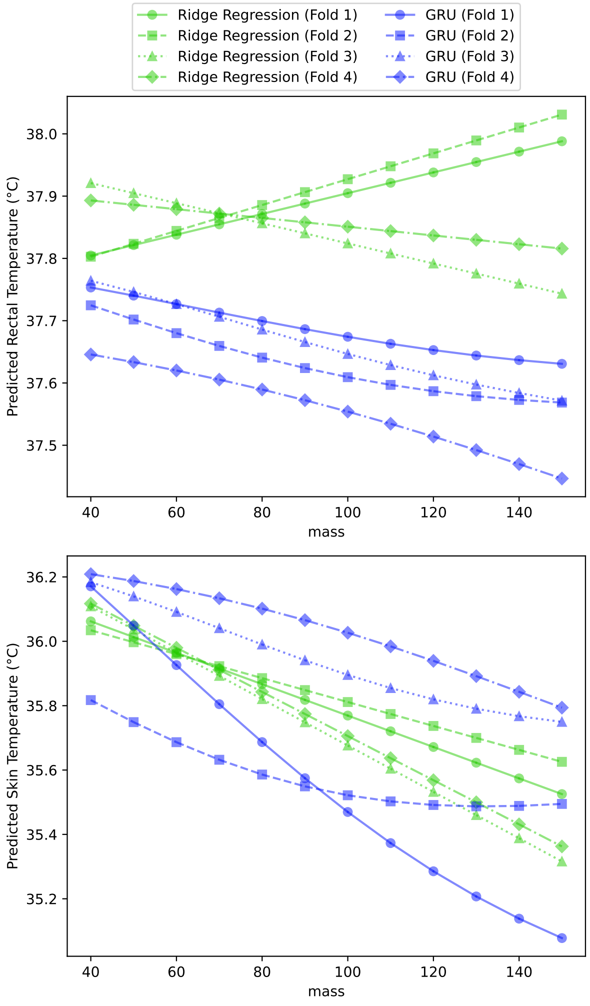

Figure S8: Predicted rectal and skin temperature over various body masses for the ML models

3.5 Ambient Temperature

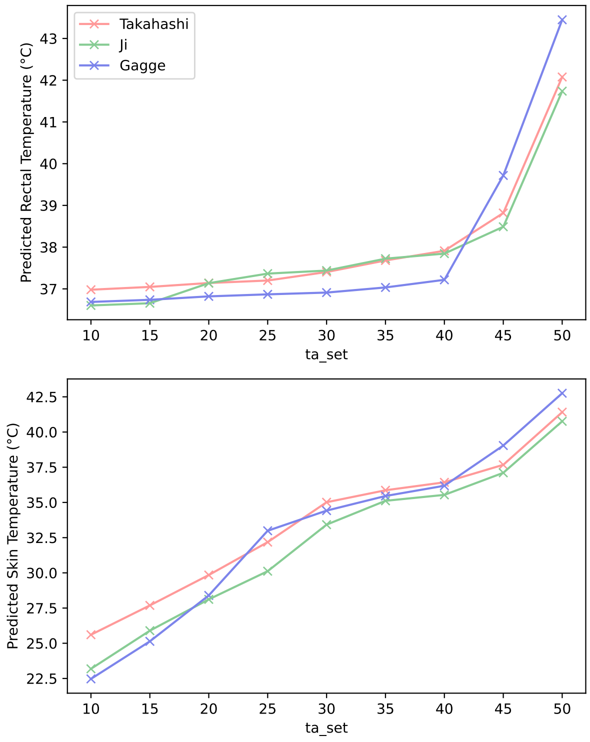

Figure S9: Predicted rectal and skin temperature over various ambient temperatures for the biophysical models

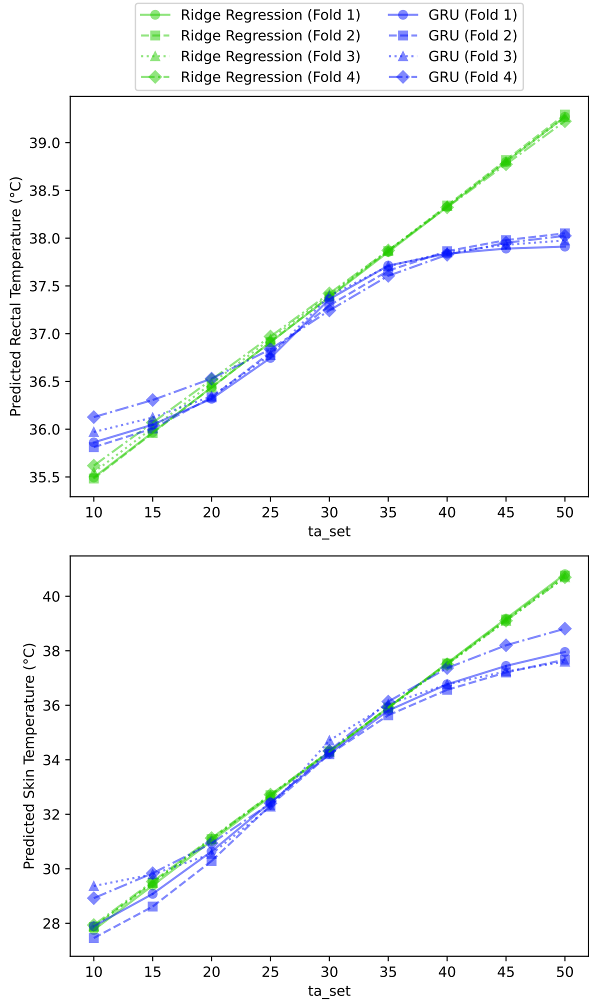

Figure S10: Predicted rectal and skin temperature over various ambient temperatures for the ML models

3.6 Relative Humidity

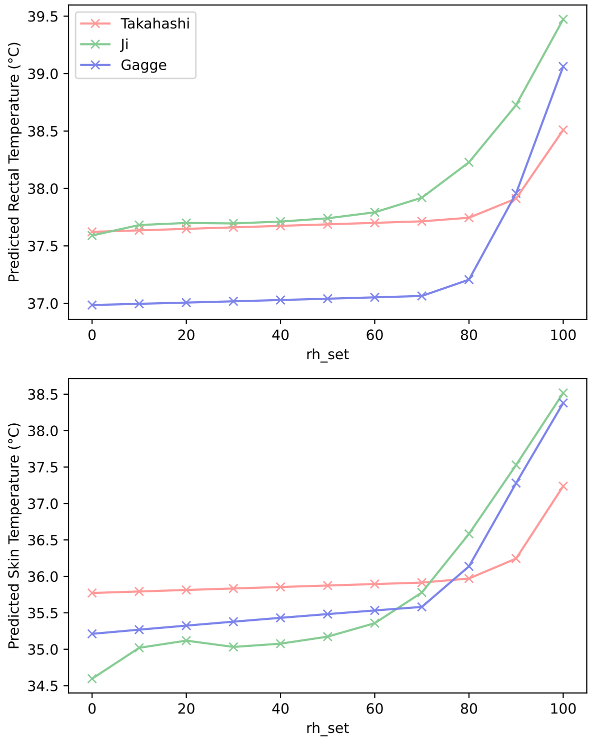

Figure S11: Predicted rectal and skin temperature over various relative humidity levels for the biophysical models

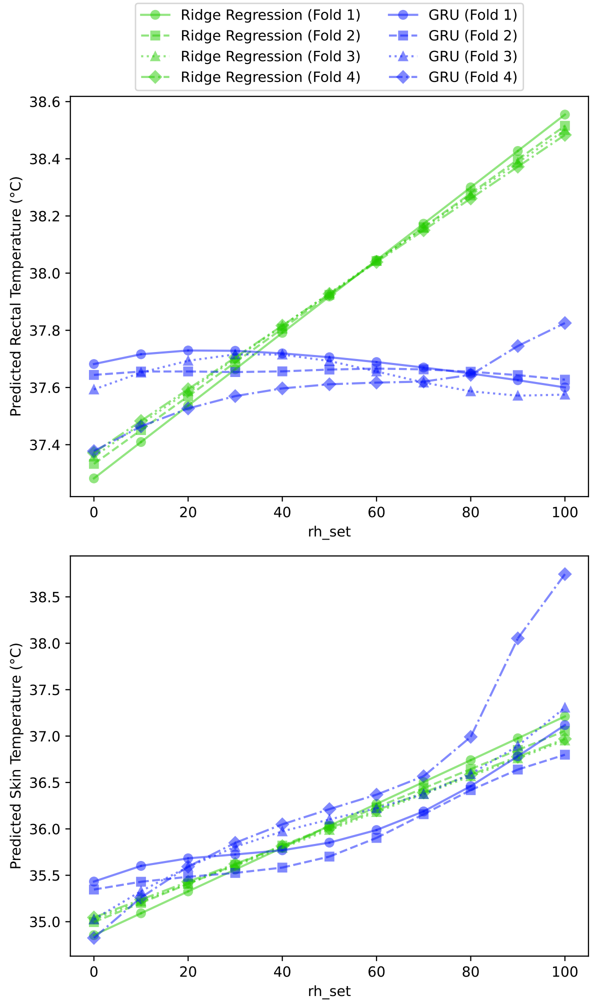

Figure S12: Predicted rectal and skin temperature over various relative humidity levels for the ML models

4 Model performance over each fold

4.1 Rectal Temperature RMSE

Table S1: Root mean squared error (RMSE) values for rectal temperature in each of the 4 cross-validation folds. All RMSE measurements are in degrees Celsius. Summary statistics (mean ± Standard Deviation) across all folds are reported in the bottom row.

Fold	ML Linear Regression	ML Ridge Regression	ML GRU	ML LSTM	ML RNN	Ji	Takahashi	Gagge
1	0.28	0.28	0.35	0.43	0.43	0.35	0.41	0.63
2	0.26	0.26	0.33	0.43	0.45	0.38	0.44	0.62
3	0.25	0.24	0.32	0.38	0.38	0.41	0.46	0.54
4	0.31	0.31	0.42	0.42	0.43	0.47	0.46	0.58
Mean ± SD	0.28 ± 0.02	0.27 ± 0.03	0.36 ± 0.04	0.42 ± 0.02	0.42 ± 0.03	0.4 ± 0.04	0.44 ± 0.02	0.59 ± 0.04

4.2 Skin Temperature RMSE

Table S2: Root mean squared error (RMSE) values for skin temperature in each of the 4 cross-validation folds. All RMSE measurements are in degrees Celsius. Summary statistics (mean ± Standard Deviation) across all folds are reported in the bottom row.

Fold	ML Linear Regression	ML Ridge Regression	ML GRU	ML LSTM	ML RNN	Ji	Takahashi	Gagge
1	0.73	0.71	0.91	1.21	1.26	1.93	0.80	0.95
2	0.73	0.71	0.94	1.06	1.16	1.76	0.71	0.83
3	0.70	0.68	0.80	1.10	1.11	1.81	0.68	0.79
4	0.83	0.81	1.27	1.09	1.12	1.90	0.76	0.89
Mean ± SD	0.75 ± 0.05	0.73 ± 0.05	0.98 ± 0.18	1.12 ± 0.06	1.16 ± 0.06	1.85 ± 0.07	0.74 ± 0.05	0.86 ± 0.06

4.3 Body Temperature RMSE

Table S3: Root mean squared error (RMSE) values for body temperature in each of the 4 cross-validation folds. All RMSE measurements are in degrees Celsius. Summary statistics (mean ± Standard Deviation) across all folds are reported in the bottom row.

Fold	ML Linear Regression	ML Ridge Regression	ML GRU	ML LSTM	ML RNN	Ji	Takahashi	Gagge

1	0.34	0.34	0.48	0.6	0.62	0.67	0.38	0.62
2	0.32	0.31	0.47	0.55	0.61	0.61	0.39	0.58
3	0.33	0.32	0.41	0.55	0.56	0.56	0.38	0.53
4	0.41	0.40	0.65	0.57	0.59	0.62	0.41	0.59
Mean ± SD	0.35 ± 0.04	0.34 ± 0.03	0.50 ± 0.09	0.57 ± 0.02	0.60 ± 0.02	0.62 ± 0.04	0.39 ± 0.01	0.58 ± 0.03

5 Individual Participant RMSE Values

5.1 Gagge

Table S4: Performance metrics for the Gagge model evaluated independently for each participant (n=76), showing Root Mean Square Error (RMSE) and Mean Bias Error (MBE) for core temperature (Tre), mean skin temperature (mTsk), and body temperature (Tb) predictions across all experimental conditions. Each row represents a single participant's results, demonstrating the model's performance for that individual's measured data. All temperatures are in degrees Celsius. Summary statistics (mean ± Standard Deviation) across all participants are reported in the bottom row.

ID	Sex	Tested under conditions	Fold number	Tre RMSE	Tre MBE	mTsk RMSE	mTsk MBE	Tb RMSE	Tb MBE
21	Male	1	3	0.65	-0.56	0.56	-0.42	0.56	-0.51
22	Male	1	2	0.55	-0.49	0.45	-0.35	0.47	-0.44
23	Male	1, 3, 4, 5, 6	4	0.57	0.11	1.01	0.12	0.63	0.11
24	Female	1, 3, 4, 5, 6	1	0.55	-0.33	1.37	-0.33	0.68	-0.33
25	Male	1	2	0.90	-0.87	1.13	-1.01	0.94	-0.92
26	Male	1, 3, 4, 5, 6	2	0.50	-0.35	0.80	0.21	0.44	-0.14
27	Female	1	3	0.43	-0.38	1.02	-0.93	0.60	-0.58
28	Male	1	1	0.65	-0.60	0.79	-0.70	0.65	-0.63
29	Male	1, 3, 4, 5, 6	4	0.57	-0.30	1.13	-0.69	0.68	-0.44
30	Male	1	3	0.53	-0.47	0.70	-0.56	0.54	-0.50
32	Male	1	2	0.34	-0.28	0.47	-0.29	0.32	-0.28
33	Male	1	4	0.93	-0.86	0.69	-0.50	0.75	-0.73
34	Female	1	2	0.59	-0.55	0.73	-0.57	0.59	-0.56
35	Male	1	2	0.45	-0.38	0.85	-0.66	0.52	-0.48
36	Female	1	4	0.53	-0.51	1.04	-0.98	0.69	-0.67
37	Female	1	3	0.57	-0.49	1.21	-1.14	0.75	-0.72
38	Female	1	4	0.70	-0.66	1.10	-1.06	0.82	-0.81
39	Male	1	4	0.69	-0.63	0.49	-0.10	0.51	-0.44
40	Female	1	4	0.57	-0.54	1.20	-0.99	0.74	-0.70
41	Male	2, 3, 4, 5, 6	4	0.36	-0.01	0.71	-0.13	0.34	-0.06
42	Female	2	3	0.51	-0.45	1.00	-0.71	0.61	-0.54
43	Male	2	1	0.77	-0.69	1.26	-1.19	0.90	-0.87
44	Male	2	4	0.55	-0.51	0.92	-0.73	0.62	-0.59

45	Female	2	2	0.67	-0.55	0.87	-0.70	0.65	-0.61
46	Male	2, 3, 4, 5, 6	3	0.33	-0.22	0.72	-0.14	0.33	-0.19
47	Female	2	3	0.51	-0.47	1.03	-0.77	0.63	-0.58
48	Male	2, 3, 4, 5, 6	2	0.28	0.04	0.69	-0.25	0.30	-0.06
49	Female	2	3	0.47	-0.46	0.89	-0.58	0.56	-0.50
50	Female	2	1	0.84	-0.75	0.91	-0.69	0.78	-0.73
52	Male	2	4	0.86	-0.82	1.32	-1.16	0.96	-0.94
53	Male	2, 3, 4, 5, 6	3	0.37	-0.11	0.66	-0.14	0.30	-0.12
54	Female	2	2	0.79	-0.72	1.07	-0.70	0.77	-0.71
55	Male	2, 3, 4, 5, 6	2	0.48	-0.33	0.74	-0.09	0.47	-0.24
56	Male	2	4	0.42	-0.37	0.70	-0.53	0.45	-0.43
57	Female	2, 3, 4, 5, 6	1	0.42	-0.18	1.04	-0.13	0.50	-0.16
58	Female	2	4	0.44	-0.40	0.62	-0.38	0.44	-0.39
59	Male	2	2	0.27	-0.17	0.84	-0.61	0.38	-0.33
60	Female	2	4	0.60	-0.50	1.00	-0.86	0.69	-0.63
61	Male	1	3	0.64	-0.56	0.81	-0.73	0.66	-0.62
62	Male	1	1	0.76	-0.68	1.46	-1.42	0.98	-0.95
63	Female	1	3	0.68	-0.65	1.07	-1.00	0.79	-0.78
64	Male	1	4	0.17	-0.08	0.35	-0.08	0.18	-0.08
65	Male	1, 3, 4	3	0.57	-0.53	0.52	-0.33	0.49	-0.46
66	Male	1	1	0.27	-0.20	1.17	-1.13	0.54	-0.54
67	Female	1, 3, 4	2	1.05	-0.99	1.10	-0.74	1.00	-0.90
68	Male	1, 3, 4	1	0.58	-0.49	0.65	-0.48	0.57	-0.48
69	Male	1	2	0.62	-0.57	0.49	-0.31	0.51	-0.48
70	Male	1, 3, 4	2	0.66	-0.62	0.50	-0.07	0.48	-0.42
71	Male	1	1	0.61	-0.54	0.75	-0.64	0.60	-0.58
72	Male	1	1	0.85	-0.80	1.15	-0.90	0.87	-0.83
73	Male	1	3	0.49	-0.45	1.03	-0.59	0.60	-0.50
74	Male	1, 3, 4	2	0.42	-0.35	0.67	-0.21	0.41	-0.30
75	Male	1, 3, 4	1	0.75	-0.73	0.66	-0.44	0.67	-0.62
76	Female	1, 3, 4	2	0.81	-0.76	0.74	-0.32	0.65	-0.60
77	Female	1, 3, 4	4	0.65	-0.61	0.62	-0.11	0.50	-0.43
78	Male	1, 3, 4	3	0.82	-0.69	0.72	-0.50	0.71	-0.62
79	Male	1	1	0.10	-0.03	0.45	-0.28	0.18	-0.12
80	Male	1	4	0.36	-0.34	0.55	-0.38	0.38	-0.35
81	Male	3, 4, 5, 6	3	0.23	-0.13	0.76	-0.04	0.30	-0.09
82	Male	3, 4, 5, 6	3	0.56	0.02	0.96	0.17	0.64	0.08
83	Female	3, 4, 5, 6	1	0.65	-0.53	0.95	-0.43	0.60	-0.49
84	Female	3, 4, 5, 6	4	0.47	-0.36	0.90	0.02	0.42	-0.22
85	Female	3, 4, 5, 6	2	0.59	-0.49	1.30	-0.41	0.69	-0.46
86	Female	3, 4, 5, 6	1	0.41	-0.34	1.02	-0.56	0.56	-0.42
87	Male	3, 4	3	0.70	-0.64	0.60	-0.40	0.62	-0.55
88	Female	3, 4	4	0.77	-0.67	0.58	-0.38	0.64	-0.56
89	Female	3, 4	4	0.76	-0.69	1.01	-0.67	0.76	-0.68
90	Female	3, 4	1	0.79	-0.72	0.64	-0.29	0.67	-0.57
91	Male	3, 4	2	0.78	-0.62	0.72	-0.52	0.72	-0.58

92	Female	4	3	0.61	-0.48	0.84	-0.51	0.65	-0.49
93	Female	4	1	0.80	-0.77	0.45	-0.02	0.55	-0.50
94	Male	3, 4	1	0.67	-0.60	0.41	-0.30	0.54	-0.49
95	Female	3, 4	1	0.70	-0.66	0.60	0.02	0.51	-0.41
96	Male	3, 4	3	0.80	-0.77	0.52	0.22	0.48	-0.41
97	Female	3, 4	1	0.81	-0.78	0.69	-0.05	0.60	-0.52
98	Female	3, 4	2	0.71	-0.69	0.69	0.05	0.51	-0.42
Mean ± SD				0.59 ± 0.19	-0.49 ± 0.24	0.83 ± 0.26	-0.48 ± 0.37	0.59 ± 0.17	-0.49 ± 0.23

5.2 Ji

Table S5: Performance metrics for the Ji model evaluated independently for each participant (n=76), showing Root Mean Square Error (RMSE) and Mean Bias Error (MBE) for core temperature (Tre), mean skin temperature (mTsk), and body temperature (Tb) predictions across all experimental conditions. Each row represents a single participant's results, demonstrating the model's performance for that individual's measured data. All temperatures are in degrees Celsius. Summary statistics (mean ± Standard Deviation) across all participants are reported in the bottom row.

ID	Sex	Tested under conditions	Fold number	Tre RMSE	Tre MBE	mTsk RMSE	mTsk MBE	Tb RMSE	Tb MBE
21	Male	1	3	0.31	-0.03	1.56	-1.45	0.60	-0.54
22	Male	1	2	0.21	0.04	1.46	-1.37	0.50	-0.47
23	Male	1, 3, 4, 5, 6	4	0.83	0.65	1.59	-1.38	0.42	-0.08
24	Female	1, 3, 4, 5, 6	1	0.44	0.21	2.19	-1.84	0.68	-0.53
25	Male	1	2	0.40	-0.34	2.14	-2.03	0.97	-0.95
26	Male	1, 3, 4, 5, 6	2	0.38	0.19	1.48	-1.29	0.44	-0.34
27	Female	1	3	0.23	0.15	2.06	-1.97	0.64	-0.61
28	Male	1	1	0.25	-0.06	1.82	-1.72	0.68	-0.66
29	Male	1, 3, 4, 5, 6	4	0.50	0.24	2.44	-2.19	0.77	-0.63
30	Male	1	3	0.25	0.06	1.71	-1.58	0.58	-0.53
32	Male	1	2	0.30	0.25	1.47	-1.32	0.38	-0.32
33	Male	1	4	0.47	-0.33	1.67	-1.52	0.79	-0.76
34	Female	1	2	0.20	-0.02	1.76	-1.61	0.65	-0.59
35	Male	1	2	0.26	0.15	1.84	-1.69	0.57	-0.51
36	Female	1	4	0.15	0.03	2.05	-1.99	0.72	-0.70
37	Female	1	3	0.30	0.05	2.23	-2.16	0.78	-0.74
38	Female	1	4	0.26	-0.13	2.15	-2.10	0.86	-0.84
39	Male	1	4	0.29	-0.10	1.29	-1.13	0.55	-0.47
40	Female	1	4	0.16	-0.01	2.19	-2.04	0.79	-0.74
41	Male	2, 3, 4, 5, 6	4	0.61	0.52	1.97	-1.69	0.39	-0.27
42	Female	2	3	0.22	0.07	2.18	-2.08	0.74	-0.70
43	Male	2	1	0.34	-0.18	2.57	-2.47	1.02	-1.00

44	Male	2	4	0.18	0.01	2.25	-2.02	0.80	-0.72
45	Female	2	2	0.34	-0.03	2.15	-2.06	0.80	-0.77
46	Male	2, 3, 4, 5, 6	3	0.37	0.32	2.01	-1.71	0.53	-0.41
47	Female	2	3	0.18	0.05	2.28	-2.14	0.79	-0.74
48	Male	2, 3, 4, 5, 6	2	0.62	0.58	2.03	-1.81	0.41	-0.28
49	Female	2	3	0.10	0.06	2.04	-1.95	0.70	-0.66
50	Female	2	1	0.42	-0.23	2.17	-2.03	0.92	-0.88
52	Male	2	4	0.38	-0.30	2.56	-2.46	1.11	-1.08
53	Male	2, 3, 4, 5, 6	3	0.53	0.43	1.92	-1.70	0.44	-0.34
54	Female	2	2	0.35	-0.20	2.21	-2.07	0.90	-0.88
55	Male	2, 3, 4, 5, 6	2	0.36	0.21	1.81	-1.65	0.53	-0.46
56	Male	2	4	0.23	0.14	1.98	-1.82	0.63	-0.56
57	Female	2, 3, 4, 5, 6	1	0.49	0.35	2.06	-1.71	0.56	-0.39
58	Female	2	4	0.18	0.12	1.92	-1.75	0.63	-0.55
59	Male	2	2	0.40	0.35	2.10	-1.90	0.57	-0.46
60	Female	2	4	0.30	0.01	2.28	-2.22	0.82	-0.79
61	Male	1	3	0.29	-0.02	1.82	-1.75	0.68	-0.64
62	Male	1	1	0.36	-0.15	2.49	-2.45	1.00	-0.97
63	Female	1	3	0.19	-0.12	2.13	-2.05	0.84	-0.82
64	Male	1	4	0.47	0.45	1.26	-1.12	0.26	-0.12
65	Male	1, 3, 4	3	0.16	0.04	1.40	-1.23	0.47	-0.42
66	Male	1	1	0.36	0.32	2.25	-2.17	0.60	-0.57
67	Female	1, 3, 4	2	0.53	-0.41	1.82	-1.61	0.94	-0.84
68	Male	1, 3, 4	1	0.32	0.08	1.50	-1.37	0.54	-0.44
69	Male	1	2	0.22	-0.04	1.50	-1.35	0.56	-0.51
70	Male	1, 3, 4	2	0.21	-0.04	1.12	-0.95	0.44	-0.37
71	Male	1	1	0.25	-0.01	1.76	-1.66	0.63	-0.60
72	Male	1	1	0.38	-0.27	2.10	-1.92	0.89	-0.86
73	Male	1	3	0.19	0.08	1.91	-1.63	0.65	-0.53
74	Male	1, 3, 4	2	0.30	0.22	1.39	-1.10	0.41	-0.26
75	Male	1, 3, 4	1	0.24	-0.16	1.46	-1.34	0.64	-0.58
76	Female	1, 3, 4	2	0.30	-0.19	1.45	-1.21	0.61	-0.56
77	Female	1, 3, 4	4	0.22	-0.04	1.26	-0.99	0.49	-0.38
78	Male	1, 3, 4	3	0.43	-0.12	1.55	-1.40	0.64	-0.58
79	Male	1	1	0.51	0.50	1.45	-1.32	0.28	-0.15
80	Male	1	4	0.22	0.19	1.52	-1.40	0.43	-0.38
81	Male	3, 4, 5, 6	3	0.44	0.42	2.12	-1.68	0.55	-0.34
82	Male	3, 4, 5, 6	3	0.76	0.57	1.55	-1.47	0.36	-0.17
83	Female	3, 4, 5, 6	1	0.33	0.02	2.40	-2.06	0.82	-0.73
84	Female	3, 4, 5, 6	4	0.31	0.18	1.96	-1.62	0.58	-0.47
85	Female	3, 4, 5, 6	2	0.29	0.05	2.48	-2.04	0.87	-0.70
86	Female	3, 4, 5, 6	1	0.27	0.20	2.47	-2.19	0.77	-0.66
87	Male	3, 4	3	0.24	-0.05	1.29	-1.23	0.50	-0.47
88	Female	3, 4	4	0.33	-0.07	1.27	-1.16	0.50	-0.46
89	Female	3, 4	4	0.28	-0.10	1.70	-1.49	0.65	-0.60
90	Female	3, 4	1	0.30	-0.13	1.18	-1.10	0.53	-0.48

91	Male	3, 4	2	0.43	-0.02	1.39	-1.34	0.58	-0.50
92	Female	4	3	0.36	0.07	1.58	-1.48	0.60	-0.49
93	Female	4	1	0.29	-0.22	1.08	-1.01	0.53	-0.50
94	Male	3, 4	1	0.25	-0.01	1.21	-1.11	0.43	-0.40
95	Female	3, 4	1	0.22	-0.07	0.98	-0.83	0.39	-0.34
96	Male	3, 4	3	0.24	-0.19	0.91	-0.63	0.42	-0.35
97	Female	3, 4	1	0.29	-0.18	1.16	-0.86	0.52	-0.43
98	Female	3, 4	2	0.19	-0.09	0.94	-0.76	0.40	-0.33
Mean ± SD				0.33 ± 0.13	0.05 ± 0.23	1.79 ± 0.43	-1.63 ± 0.43	0.63 ± 0.18	-0.55 ± 0.21

5.3 Takahashi

Table S6: Performance metrics for the Takahashi model evaluated independently for each participant (n=76), showing Root Mean Square Error (RMSE) and Mean Bias Error (MBE) for core temperature (Tre), mean skin temperature (mTsk), and body temperature (Tb) predictions across all experimental conditions. Each row represents a single participant's results, demonstrating the model's performance for that individual's measured data. All temperatures are in degrees Celsius. Summary statistics (mean ± Standard Deviation) across all participants are reported in the bottom row.

ID	Sex	Tested under conditions	Fold number	Tre RMSE	Tre MBE	mTsk RMSE	mTsk MBE	Tb RMSE	Tb MBE
21	Male	1	3	0.28	0.13	0.41	-0.04	0.22	0.07
22	Male	1	2	0.20	0.15	0.35	0.02	0.17	0.11
23	Male	1, 3, 4, 5, 6	4	0.60	0.42	0.78	0.16	0.56	0.32
24	Female	1, 3, 4, 5, 6	1	0.57	0.44	1.16	-0.10	0.52	0.25
25	Male	1	2	0.20	-0.16	0.82	-0.63	0.37	-0.33
26	Male	1, 3, 4, 5, 6	2	0.48	0.40	0.66	0.37	0.46	0.39
27	Female	1	3	0.54	0.53	0.66	-0.49	0.22	0.16
28	Male	1	1	0.20	0.13	0.51	-0.31	0.14	-0.03
29	Male	1, 3, 4, 5, 6	4	0.38	0.08	1.03	-0.65	0.44	-0.18
30	Male	1	3	0.32	0.26	0.50	-0.15	0.22	0.11
32	Male	1	2	0.67	0.66	0.48	0.19	0.51	0.49
33	Male	1	4	0.31	-0.10	0.50	-0.10	0.22	-0.10
34	Female	1	2	0.41	0.39	0.54	-0.11	0.29	0.21
35	Male	1	2	0.43	0.40	0.63	-0.24	0.27	0.17
36	Female	1	4	0.19	0.16	0.75	-0.66	0.19	-0.14
37	Female	1	3	0.44	0.38	0.85	-0.74	0.20	-0.02
38	Female	1	4	0.27	0.22	0.70	-0.63	0.17	-0.09
39	Male	1	4	0.35	0.28	0.64	0.36	0.39	0.31
40	Female	1	4	0.34	0.33	0.88	-0.54	0.25	0.01
41	Male	2, 3, 4, 5, 6	4	0.72	0.66	0.67	0.02	0.48	0.43
42	Female	2	3	0.34	0.30	0.75	-0.30	0.28	0.08

43	Male	2	1	0.26	0.14	0.84	-0.70	0.24	-0.17
44	Male	2	4	0.11	-0.01	0.62	-0.35	0.23	-0.13
45	Female	2	2	0.38	0.28	0.57	-0.26	0.22	0.08
46	Male	2, 3, 4, 5, 6	3	0.44	0.41	0.68	0.03	0.35	0.27
47	Female	2	3	0.32	0.30	0.77	-0.32	0.28	0.08
48	Male	2, 3, 4, 5, 6	2	0.70	0.67	0.61	-0.06	0.47	0.41
49	Female	2	3	0.33	0.32	0.72	-0.13	0.31	0.16
50	Female	2	1	0.31	0.09	0.67	-0.23	0.29	-0.02
52	Male	2	4	0.35	-0.32	1.04	-0.84	0.54	-0.51
53	Male	2, 3, 4, 5, 6	3	0.53	0.45	0.63	-0.04	0.35	0.27
54	Female	2	2	0.19	-0.04	0.86	-0.28	0.31	-0.13
55	Male	2, 3, 4, 5, 6	2	0.55	0.48	0.54	0.10	0.42	0.35
56	Male	2	4	0.30	0.27	0.50	-0.14	0.21	0.12
57	Female	2, 3, 4, 5, 6	1	0.61	0.55	0.91	0.11	0.52	0.39
58	Female	2	4	0.35	0.33	0.52	0.06	0.31	0.23
59	Male	2	2	0.62	0.60	0.61	-0.16	0.40	0.33
60	Female	2	4	0.51	0.46	0.62	-0.36	0.30	0.16
61	Male	1	3	0.21	-0.02	0.55	-0.41	0.24	-0.16
62	Male	1	1	0.25	0.02	1.11	-1.05	0.41	-0.37
63	Female	1	3	0.23	0.21	0.70	-0.56	0.16	-0.07
64	Male	1	4	0.85	0.84	0.57	0.40	0.71	0.68
65	Male	1, 3, 4	3	0.28	0.25	0.49	0.14	0.26	0.21
66	Male	1	1	0.77	0.76	0.73	-0.62	0.29	0.26
67	Female	1, 3, 4	2	0.38	-0.26	0.90	-0.27	0.49	-0.26
68	Male	1, 3, 4	1	0.32	0.24	0.45	-0.04	0.27	0.14
69	Male	1	2	0.31	0.27	0.46	0.14	0.28	0.22
70	Male	1, 3, 4	2	0.24	0.02	0.64	0.37	0.26	0.15
71	Male	1	1	0.19	0.09	0.51	-0.31	0.14	-0.05
72	Male	1	1	0.24	-0.13	0.91	-0.54	0.37	-0.28
73	Male	1	3	0.47	0.46	0.89	-0.13	0.41	0.25
74	Male	1, 3, 4	2	0.41	0.39	0.72	0.24	0.41	0.33
75	Male	1, 3, 4	1	0.12	-0.00	0.52	0.03	0.21	0.01
76	Female	1, 3, 4	2	0.23	-0.00	0.68	0.13	0.22	0.04
77	Female	1, 3, 4	4	0.24	0.18	0.71	0.39	0.34	0.26
78	Male	1, 3, 4	3	0.38	0.12	0.58	-0.02	0.29	0.07
79	Male	1	1	0.74	0.72	0.44	0.13	0.54	0.51
80	Male	1	4	0.35	0.34	0.43	0.01	0.27	0.22
81	Male	3, 4, 5, 6	3	0.64	0.63	0.89	0.06	0.52	0.43
82	Male	3, 4, 5, 6	3	0.66	0.48	0.57	0.16	0.56	0.36
83	Female	3, 4, 5, 6	1	0.32	0.10	0.83	-0.24	0.28	-0.02
84	Female	3, 4, 5, 6	4	0.38	0.30	0.82	0.20	0.37	0.26
85	Female	3, 4, 5, 6	2	0.27	0.09	1.12	-0.19	0.43	-0.01
86	Female	3, 4, 5, 6	1	0.31	0.28	0.83	-0.37	0.28	0.05
87	Male	3, 4	3	0.34	0.28	0.43	0.16	0.31	0.24
88	Female	3, 4	4	0.28	0.08	0.47	0.12	0.26	0.09
89	Female	3, 4	4	0.29	0.19	0.81	-0.13	0.30	0.07

90	Female	3, 4	1	0.24	0.08	0.59	0.22	0.32	0.13
91	Male	3, 4	2	0.39	0.12	0.41	-0.05	0.33	0.06
92	Female	4	3	0.37	0.24	0.58	0.08	0.38	0.18
93	Female	4	1	0.15	-0.07	0.69	0.60	0.22	0.17
94	Male	3, 4	1	0.20	0.01	0.32	0.16	0.17	0.06
95	Female	3, 4	1	0.47	0.43	0.88	0.68	0.57	0.52
96	Male	3, 4	3	0.35	0.32	1.02	0.89	0.56	0.52
97	Female	3, 4	1	0.22	0.11	0.87	0.50	0.38	0.25
98	Female	3, 4	2	0.18	0.13	0.90	0.61	0.39	0.30
Mean ± SD				0.37 ± 0.17	0.25 ± 0.24	0.68 ± 0.19	-0.09 ± 0.37	0.34 ± 0.12	0.13 ± 0.22

5.4 ML Ridge Regression

Table S7: Performance metrics for the ML ridge regression model evaluated independently for each participant (n=76), showing Root Mean Square Error (RMSE) and Mean Bias Error (MBE) for core temperature (Tre), mean skin temperature (mTsk), and body temperature (Tb) predictions across all experimental conditions. Each row represents a single participant's results, demonstrating the model's performance for that individual's measured data. All temperatures are in degrees Celsius. Summary statistics (mean ± Standard Deviation) across all participants are reported in the bottom row.

ID	Sex	Tested under conditions	Fold number	Tre RMSE	Tre MBE	mTsk RMSE	mTsk MBE	Tb RMSE	Tb MBE
21	Male	1	3	0.14	-0.11	0.67	0.22	0.22	0.01
22	Male	1	2	0.08	-0.04	0.64	0.26	0.23	0.07
23	Male	1, 3, 4, 5, 6	4	0.54	0.44	1.04	0.47	0.65	0.45
24	Female	1, 3, 4, 5, 6	1	0.28	0.08	0.90	0.06	0.37	0.07
25	Male	1	2	0.39	-0.37	0.81	-0.33	0.45	-0.35
26	Male	1, 3, 4, 5, 6	2	0.16	-0.04	0.65	0.38	0.23	0.11
27	Female	1	3	0.27	0.23	0.73	-0.08	0.32	0.12
28	Male	1	1	0.17	-0.11	0.70	-0.09	0.26	-0.10
29	Male	1, 3, 4, 5, 6	4	0.32	0.05	0.74	-0.37	0.38	-0.10
30	Male	1	3	0.10	0.01	0.72	0.07	0.26	0.03
32	Male	1	2	0.23	0.19	0.76	0.24	0.36	0.21
33	Male	1	4	0.40	-0.37	0.80	0.17	0.34	-0.17
34	Female	1	2	0.13	0.09	0.83	0.24	0.33	0.14
35	Male	1	2	0.19	0.15	0.80	0.00	0.30	0.09
36	Female	1	4	0.29	0.15	0.62	0.02	0.30	0.10
37	Female	1	3	0.18	0.14	0.68	-0.15	0.24	0.03
38	Female	1	4	0.10	-0.01	0.62	-0.22	0.26	-0.09
39	Male	1	4	0.15	-0.13	0.81	0.47	0.27	0.09
40	Female	1	4	0.14	0.01	0.93	-0.32	0.37	-0.11
41	Male	2, 3, 4, 5, 6	4	0.32	0.24	0.63	-0.00	0.32	0.15

42	Female	2	3	0.19	-0.01	0.88	-0.24	0.34	-0.09
43	Male	2	1	0.46	-0.42	1.22	-0.92	0.63	-0.60
44	Male	2	4	0.28	-0.24	1.11	-0.38	0.45	-0.29
45	Female	2	2	0.23	-0.11	0.78	-0.26	0.27	-0.16
46	Male	2, 3, 4, 5, 6	3	0.13	0.02	0.65	-0.04	0.27	-0.00
47	Female	2	3	0.14	-0.08	0.97	-0.35	0.40	-0.18
48	Male	2, 3, 4, 5, 6	2	0.31	0.26	0.62	-0.19	0.28	0.10
49	Female	2	3	0.18	-0.08	0.73	-0.14	0.32	-0.10
50	Female	2	1	0.47	-0.38	1.01	-0.46	0.51	-0.40
52	Male	2	4	0.48	-0.47	1.11	-0.71	0.63	-0.56
53	Male	2, 3, 4, 5, 6	3	0.28	0.18	0.52	0.02	0.26	0.12
54	Female	2	2	0.39	-0.34	0.99	-0.21	0.42	-0.29
55	Male	2, 3, 4, 5, 6	2	0.17	-0.08	0.62	-0.08	0.28	-0.08
56	Male	2	4	0.14	-0.06	0.87	-0.13	0.34	-0.08
57	Female	2, 3, 4, 5, 6	1	0.29	0.20	0.75	0.22	0.40	0.21
58	Female	2	4	0.13	-0.01	0.92	0.07	0.34	0.02
59	Male	2	2	0.20	0.13	1.03	-0.29	0.38	-0.02
60	Female	2	4	0.20	-0.10	0.82	-0.50	0.32	-0.24
61	Male	1	3	0.14	-0.07	0.58	-0.01	0.21	-0.05
62	Male	1	1	0.21	-0.17	0.93	-0.75	0.41	-0.38
63	Female	1	3	0.21	-0.08	0.71	-0.28	0.33	-0.15
64	Male	1	4	0.41	0.38	0.74	0.38	0.48	0.38
65	Male	1, 3, 4	3	0.12	-0.01	0.61	-0.03	0.26	-0.02
66	Male	1	1	0.29	0.25	0.95	-0.69	0.29	-0.09
67	Female	1, 3, 4	2	0.42	-0.39	0.70	-0.14	0.41	-0.30
68	Male	1, 3, 4	1	0.19	0.01	0.53	-0.23	0.23	-0.07
69	Male	1	2	0.13	-0.11	0.75	0.21	0.28	0.01
70	Male	1, 3, 4	2	0.29	-0.19	0.52	0.23	0.24	-0.04
71	Male	1	1	0.12	-0.04	0.70	0.02	0.23	-0.02
72	Male	1	1	0.39	-0.30	0.98	-0.21	0.45	-0.26
73	Male	1	3	0.11	0.04	1.05	-0.08	0.39	-0.01
74	Male	1, 3, 4	2	0.19	0.15	0.60	0.09	0.28	0.13
75	Male	1, 3, 4	1	0.32	-0.27	0.44	-0.15	0.31	-0.22
76	Female	1, 3, 4	2	0.27	-0.22	0.75	0.10	0.29	-0.11
77	Female	1, 3, 4	4	0.18	-0.05	0.94	0.44	0.39	0.13
78	Male	1, 3, 4	3	0.30	-0.20	0.60	-0.23	0.27	-0.21
79	Male	1	1	0.51	0.44	0.72	0.28	0.53	0.39
80	Male	1	4	0.20	0.11	0.71	0.26	0.34	0.16
81	Male	3, 4, 5, 6	3	0.23	0.11	0.58	-0.04	0.31	0.06
82	Male	3, 4, 5, 6	3	0.41	0.29	0.87	0.37	0.54	0.32
83	Female	3, 4, 5, 6	1	0.27	-0.15	0.57	-0.05	0.29	-0.12
84	Female	3, 4, 5, 6	4	0.16	-0.02	0.59	0.26	0.24	0.08
85	Female	3, 4, 5, 6	2	0.27	-0.13	0.90	-0.05	0.41	-0.10
86	Female	3, 4, 5, 6	1	0.20	0.05	0.60	-0.16	0.27	-0.03
87	Male	3, 4	3	0.22	-0.17	0.50	-0.37	0.29	-0.24
88	Female	3, 4	4	0.16	-0.05	0.47	0.01	0.15	-0.03

89	Female	3, 4	4	0.19	-0.08	0.79	-0.44	0.33	-0.21
90	Female	3, 4	1	0.17	-0.12	0.44	0.02	0.20	-0.07
91	Male	3, 4	2	0.29	-0.13	0.61	-0.45	0.35	-0.25
92	Female	4	3	0.21	0.04	0.73	-0.42	0.32	-0.12
93	Female	4	1	0.35	-0.33	0.37	-0.06	0.27	-0.24
94	Male	3, 4	1	0.18	-0.15	0.45	-0.17	0.21	-0.16
95	Female	3, 4	1	0.16	-0.13	0.52	0.09	0.20	-0.05
96	Male	3, 4	3	0.37	-0.35	0.54	-0.03	0.33	-0.24
97	Female	3, 4	1	0.24	-0.18	0.75	0.25	0.29	-0.02
98	Female	3, 4	2	0.19	-0.12	0.65	0.32	0.21	0.04
Mean ± SD				0.24 ± 0.11	-0.04 ± 0.20	0.74 ± 0.18	-0.07 ± 0.29	0.33 ± 0.10	-0.05 ± 0.19

5.5 ML Linear Regression

Table S8: Performance metrics for the ML linear regression model evaluated independently for each participant (n=76), showing Root Mean Square Error (RMSE) and Mean Bias Error (MBE) for core temperature (Tre), mean skin temperature (mTsk), and body temperature (Tb) predictions across all experimental conditions. Each row represents a single participant's results, demonstrating the model's performance for that individual's measured data. All temperatures are in degrees Celsius. Summary statistics (mean ± Standard Deviation) across all participants are reported in the bottom row.

ID	Sex	Tested under conditions	Fold number	Tre RMSE	Tre MBE	mTsk RMSE	mTsk MBE	Tb RMSE	Tb MBE
21	Male	1	3	0.14	-0.11	0.70	0.20	0.23	0.00
22	Male	1	2	0.11	-0.03	0.67	0.24	0.24	0.07
23	Male	1, 3, 4, 5, 6	4	0.54	0.44	1.04	0.46	0.65	0.45
24	Female	1, 3, 4, 5, 6	1	0.28	0.07	0.91	0.06	0.38	0.07
25	Male	1	2	0.40	-0.37	0.84	-0.34	0.47	-0.36
26	Male	1, 3, 4, 5, 6	2	0.16	-0.05	0.66	0.38	0.23	0.11
27	Female	1	3	0.27	0.22	0.76	-0.08	0.33	0.11
28	Male	1	1	0.19	-0.10	0.74	-0.11	0.28	-0.11
29	Male	1, 3, 4, 5, 6	4	0.32	0.05	0.75	-0.38	0.38	-0.10
30	Male	1	3	0.10	0.01	0.75	0.06	0.27	0.03
32	Male	1	2	0.24	0.19	0.79	0.22	0.37	0.20
33	Male	1	4	0.40	-0.37	0.83	0.16	0.35	-0.18
34	Female	1	2	0.15	0.08	0.86	0.23	0.33	0.13
35	Male	1	2	0.20	0.14	0.84	-0.01	0.31	0.09
36	Female	1	4	0.31	0.14	0.65	0.01	0.32	0.09
37	Female	1	3	0.18	0.12	0.71	-0.15	0.25	0.02
38	Female	1	4	0.12	-0.02	0.66	-0.23	0.27	-0.10
39	Male	1	4	0.15	-0.14	0.82	0.46	0.27	0.08
40	Female	1	4	0.16	0.00	0.96	-0.33	0.39	-0.12

41	Male	2, 3, 4, 5, 6	4	0.32	0.23	0.67	-0.01	0.33	0.15
42	Female	2	3	0.19	-0.01	0.96	-0.26	0.36	-0.10
43	Male	2	1	0.47	-0.42	1.28	-0.94	0.65	-0.61
44	Male	2	4	0.28	-0.23	1.17	-0.41	0.46	-0.30
45	Female	2	2	0.25	-0.11	0.85	-0.28	0.28	-0.17
46	Male	2, 3, 4, 5, 6	3	0.13	0.01	0.69	-0.04	0.27	-0.01
47	Female	2	3	0.15	-0.09	1.04	-0.37	0.42	-0.19
48	Male	2, 3, 4, 5, 6	2	0.30	0.26	0.65	-0.19	0.28	0.10
49	Female	2	3	0.19	-0.09	0.79	-0.16	0.34	-0.11
50	Female	2	1	0.47	-0.37	1.09	-0.48	0.52	-0.41
52	Male	2	4	0.47	-0.47	1.18	-0.73	0.64	-0.56
53	Male	2, 3, 4, 5, 6	3	0.27	0.18	0.55	0.02	0.26	0.12
54	Female	2	2	0.41	-0.34	1.07	-0.22	0.44	-0.30
55	Male	2, 3, 4, 5, 6	2	0.17	-0.08	0.65	-0.09	0.29	-0.08
56	Male	2	4	0.14	-0.06	0.94	-0.15	0.35	-0.09
57	Female	2, 3, 4, 5, 6	1	0.28	0.19	0.77	0.22	0.40	0.20
58	Female	2	4	0.13	-0.01	0.99	0.05	0.36	0.01
59	Male	2	2	0.19	0.13	1.10	-0.31	0.39	-0.03
60	Female	2	4	0.21	-0.11	0.87	-0.51	0.33	-0.25
61	Male	1	3	0.15	-0.08	0.61	-0.03	0.22	-0.06
62	Male	1	1	0.22	-0.18	0.96	-0.76	0.42	-0.39
63	Female	1	3	0.24	-0.10	0.74	-0.28	0.35	-0.16
64	Male	1	4	0.40	0.37	0.76	0.37	0.48	0.37
65	Male	1, 3, 4	3	0.14	-0.02	0.63	-0.03	0.27	-0.02
66	Male	1	1	0.29	0.24	0.98	-0.70	0.31	-0.10
67	Female	1, 3, 4	2	0.43	-0.41	0.71	-0.13	0.42	-0.31
68	Male	1, 3, 4	1	0.19	0.01	0.55	-0.23	0.23	-0.08
69	Male	1	2	0.15	-0.11	0.78	0.20	0.29	-0.00
70	Male	1, 3, 4	2	0.31	-0.20	0.54	0.23	0.24	-0.05
71	Male	1	1	0.14	-0.04	0.73	0.00	0.25	-0.02
72	Male	1	1	0.41	-0.30	1.01	-0.22	0.46	-0.27
73	Male	1	3	0.13	0.04	1.08	-0.10	0.40	-0.01
74	Male	1, 3, 4	2	0.19	0.13	0.63	0.09	0.28	0.12
75	Male	1, 3, 4	1	0.33	-0.28	0.45	-0.15	0.32	-0.23
76	Female	1, 3, 4	2	0.28	-0.24	0.77	0.10	0.30	-0.11
77	Female	1, 3, 4	4	0.19	-0.07	0.96	0.45	0.39	0.12
78	Male	1, 3, 4	3	0.30	-0.21	0.63	-0.23	0.28	-0.22
79	Male	1	1	0.52	0.44	0.75	0.27	0.53	0.38
80	Male	1	4	0.21	0.10	0.74	0.25	0.35	0.15
81	Male	3, 4, 5, 6	3	0.23	0.11	0.58	-0.04	0.31	0.05
82	Male	3, 4, 5, 6	3	0.41	0.29	0.87	0.37	0.54	0.32
83	Female	3, 4, 5, 6	1	0.28	-0.16	0.58	-0.05	0.29	-0.12
84	Female	3, 4, 5, 6	4	0.16	-0.03	0.60	0.27	0.24	0.08
85	Female	3, 4, 5, 6	2	0.27	-0.14	0.90	-0.04	0.42	-0.11
86	Female	3, 4, 5, 6	1	0.20	0.04	0.60	-0.16	0.27	-0.03
87	Male	3, 4	3	0.23	-0.19	0.50	-0.36	0.30	-0.25

88	Female	3, 4	4	0.17	-0.06	0.48	0.02	0.15	-0.03
89	Female	3, 4	4	0.21	-0.09	0.79	-0.42	0.34	-0.21
90	Female	3, 4	1	0.18	-0.13	0.43	0.03	0.20	-0.07
91	Male	3, 4	2	0.29	-0.14	0.60	-0.44	0.34	-0.25
92	Female	4	3	0.20	0.03	0.72	-0.41	0.32	-0.13
93	Female	4	1	0.36	-0.35	0.37	-0.05	0.27	-0.24
94	Male	3, 4	1	0.19	-0.17	0.46	-0.16	0.22	-0.16
95	Female	3, 4	1	0.19	-0.16	0.53	0.11	0.20	-0.06
96	Male	3, 4	3	0.38	-0.37	0.56	-0.02	0.34	-0.25
97	Female	3, 4	1	0.26	-0.20	0.76	0.27	0.30	-0.03
98	Female	3, 4	2	0.21	-0.14	0.66	0.34	0.21	0.03
Mean ± SD				0.25 ± 0.11	-0.05 ± 0.20	0.77 ± 0.19	-0.07 ± 0.30	0.34 ± 0.10	-0.06 ± 0.19

5.6 ML GRU

Table S9: Performance metrics for the ML GRU model evaluated independently for each participant (n=76), showing Root Mean Square Error (RMSE) and Mean Bias Error (MBE) for core temperature (Tre), mean skin temperature (mTsk), and body temperature (Tb) predictions across all experimental conditions. Each row represents a single participant's results, demonstrating the model's performance for that individual's measured data. All temperatures are in degrees Celsius. Summary statistics (mean ± Standard Deviation) across all participants are reported in the bottom row.

ID	Sex	Tested under conditions	Fold number	Tre RMSE	Tre MBE	mTsk RMSE	mTsk MBE	Tb RMSE	Tb MBE
21	Male	1	3	0.19	-0.03	0.31	0.02	0.17	-0.01
22	Male	1	2	0.11	-0.10	0.37	-0.31	0.19	-0.18
23	Male	1, 3, 4, 5, 6	4	0.47	0.35	0.88	0.18	0.52	0.29
24	Female	1, 3, 4, 5, 6	1	0.34	0.05	1.15	0.15	0.52	0.09
25	Male	1	2	0.34	-0.33	0.76	-0.58	0.46	-0.42
26	Male	1, 3, 4, 5, 6	2	0.21	-0.16	0.74	0.40	0.25	0.04
27	Female	1	3	0.30	0.29	0.67	-0.43	0.18	0.03
28	Male	1	1	0.26	-0.22	0.62	-0.50	0.35	-0.32
29	Male	1, 3, 4, 5, 6	4	0.36	-0.07	1.00	-0.65	0.50	-0.28
30	Male	1	3	0.15	0.07	0.40	-0.11	0.16	0.01
32	Male	1	2	0.18	0.15	0.32	-0.11	0.15	0.06
33	Male	1	4	0.41	-0.32	0.80	0.30	0.24	-0.09
34	Female	1	2	0.08	-0.02	0.58	-0.30	0.23	-0.12
35	Male	1	2	0.20	0.17	0.60	-0.24	0.22	0.02
36	Female	1	4	0.21	0.17	0.55	0.13	0.25	0.16
37	Female	1	3	0.34	0.26	0.57	-0.34	0.22	0.04
38	Female	1	4	0.14	-0.04	0.56	-0.12	0.18	-0.07
39	Male	1	4	0.21	-0.12	0.84	0.61	0.24	0.14

40	Female	1	4	0.10	-0.03	0.90	-0.37	0.32	-0.16
41	Male	2, 3, 4, 5, 6	4	0.36	0.11	1.17	-0.33	0.56	-0.05
42	Female	2	3	0.42	-0.08	1.43	-1.13	0.70	-0.46
43	Male	2	1	0.85	-0.68	2.74	-1.96	1.51	-1.14
44	Male	2	4	0.72	-0.42	2.81	-1.48	1.45	-0.80
45	Female	2	2	0.53	-0.27	1.52	-1.12	0.85	-0.58
46	Male	2, 3, 4, 5, 6	3	0.31	-0.11	1.09	-0.27	0.55	-0.17
47	Female	2	3	0.47	-0.12	1.50	-1.13	0.78	-0.48
48	Male	2, 3, 4, 5, 6	2	0.28	0.16	1.03	-0.14	0.49	0.05
49	Female	2	3	0.45	-0.10	1.28	-0.86	0.70	-0.37
50	Female	2	1	0.73	-0.63	1.34	-1.05	0.90	-0.78
52	Male	2	4	0.99	-0.67	2.62	-1.80	1.56	-1.08
53	Male	2, 3, 4, 5, 6	3	0.35	0.02	0.79	-0.38	0.44	-0.13
54	Female	2	2	0.57	-0.46	1.41	-1.02	0.84	-0.66
55	Male	2, 3, 4, 5, 6	2	0.34	-0.22	1.22	0.05	0.59	-0.12
56	Male	2	4	0.72	-0.25	2.50	-1.20	1.34	-0.59
57	Female	2, 3, 4, 5, 6	1	0.38	0.14	0.96	0.23	0.55	0.17
58	Female	2	4	0.64	-0.18	2.32	-0.93	1.23	-0.45
59	Male	2	2	0.54	-0.07	2.42	-1.34	1.18	-0.53
60	Female	2	4	0.71	-0.30	2.50	-1.47	1.32	-0.73
61	Male	1	3	0.16	-0.00	0.39	-0.24	0.18	-0.09
62	Male	1	1	0.23	-0.17	1.00	-0.95	0.47	-0.45
63	Female	1	3	0.12	-0.06	0.83	-0.71	0.34	-0.30
64	Male	1	4	0.38	0.37	0.64	0.45	0.44	0.40
65	Male	1, 3, 4	3	0.08	-0.03	0.48	0.05	0.16	-0.00
66	Male	1	1	0.20	0.18	1.16	-1.12	0.32	-0.29
67	Female	1, 3, 4	2	0.49	-0.43	0.86	-0.41	0.56	-0.42
68	Male	1, 3, 4	1	0.24	-0.16	0.70	-0.58	0.36	-0.31
69	Male	1	2	0.17	-0.13	0.30	-0.11	0.18	-0.13
70	Male	1, 3, 4	2	0.33	-0.23	0.58	0.18	0.22	-0.08
71	Male	1	1	0.14	-0.09	0.49	-0.27	0.21	-0.16
72	Male	1	1	0.34	-0.28	0.83	-0.38	0.43	-0.31
73	Male	1	3	0.05	-0.01	0.94	-0.46	0.34	-0.17
74	Male	1, 3, 4	2	0.13	0.05	0.59	-0.00	0.21	0.03
75	Male	1, 3, 4	1	0.34	-0.33	0.49	-0.28	0.35	-0.31
76	Female	1, 3, 4	2	0.34	-0.31	0.66	-0.05	0.30	-0.21
77	Female	1, 3, 4	4	0.18	-0.05	1.03	0.62	0.37	0.19
78	Male	1, 3, 4	3	0.42	-0.26	0.54	-0.29	0.36	-0.27
79	Male	1	1	0.43	0.38	0.37	-0.07	0.32	0.22
80	Male	1	4	0.17	0.17	0.64	0.33	0.30	0.22
81	Male	3, 4, 5, 6	3	0.20	-0.03	0.59	-0.08	0.30	-0.05
82	Male	3, 4, 5, 6	3	0.33	0.18	0.70	0.23	0.41	0.20
83	Female	3, 4, 5, 6	1	0.30	-0.19	0.58	0.10	0.30	-0.08
84	Female	3, 4, 5, 6	4	0.20	-0.09	0.69	0.38	0.23	0.08
85	Female	3, 4, 5, 6	2	0.29	-0.17	0.96	-0.21	0.45	-0.18
86	Female	3, 4, 5, 6	1	0.18	0.00	0.65	-0.04	0.27	-0.01

87	Male	3, 4	3	0.33	-0.27	0.55	-0.24	0.35	-0.26
88	Female	3, 4	4	0.32	-0.16	0.54	-0.00	0.21	-0.11
89	Female	3, 4	4	0.32	-0.23	0.93	-0.40	0.41	-0.29
90	Female	3, 4	1	0.33	-0.24	0.66	-0.15	0.39	-0.21
91	Male	3, 4	2	0.45	-0.32	0.71	-0.47	0.50	-0.38
92	Female	4	3	0.27	0.05	0.80	-0.56	0.33	-0.17
93	Female	4	1	0.31	-0.28	0.66	0.23	0.27	-0.10
94	Male	3, 4	1	0.30	-0.25	0.41	-0.23	0.28	-0.24
95	Female	3, 4	1	0.23	-0.15	0.74	0.08	0.33	-0.07
96	Male	3, 4	3	0.51	-0.49	0.60	0.20	0.35	-0.24
97	Female	3, 4	1	0.30	-0.26	0.75	0.12	0.32	-0.12
98	Female	3, 4	2	0.21	-0.17	0.76	0.19	0.26	-0.04
Mean ± SD				0.33 ± 0.18	-0.11 ± 0.22	0.94 ± 0.59	-0.33 ± 0.55	0.46 ± 0.34	-0.19 ± 0.29
